# Cellular effects of terahertz waves

**DOI:** 10.1117/1.JBO.26.9.090902

**Published:** 2021-09-30

**Authors:** Olga P. Cherkasova, Danil S. Serdyukov, Eugenia F. Nemova, Alexander S. Ratushnyak, Anna S. Kucheryavenko, Irina N. Dolganova, Guofu Xu, Maksim Skorobogatiy, Igor V. Reshetov, Peter S. Timashev, Igor E. Spektor, Kirill I. Zaytsev, Valery V. Tuchin

**Affiliations:** a Institute of Laser Physics of the Siberian Branch of the Russian Academy of Sciences, Russian Federation; b Novosibirsk State Technical University, Russian Federation; c Federal Research Center Institute of Cytology and Genetics of the Siberian Branch of the Russian Academy of Sciences, Russian Federation; d Institute of Computational Technologies of the Siberian Branch of the Russian Academy of Sciences, Russian Federation; e Institute of Solid State Physics of the Russian Academy of Sciences, Russian Federation; f Prokhorov General Physics Institute of the Russian Academy of Sciences, Russian Federation; g Sechenov University, Institute for Regenerative Medicine, Russian Federation; h Sechenov University, World-Class Research Center “Digital Biodesign and Personalized Healthcare,” Russian Federation; i Polytechnique Montreal, Department of Engineering Physics, Canada; j Sechenov University, Institute for Cluster Oncology, Russian Federation; k Academy of Postgraduate Education FSCC FMBA, Russian Federation; l N.N. Semenov Institute of Chemical Physics, Department of Polymers and Composites, Russian Federation; m Lomonosov Moscow State University, Department of Chemistry, Russian Federation; n Bauman Moscow State Technical University, Russian Federation; o Saratov State University, Russian Federation; p Institute of Precision Mechanics and Control of the Russian Academy of Sciences, Russian Federation; q National Research Tomsk State University, Russian Federation

**Keywords:** THz technology, THz biophotonics, THz medical diagnosis and therapy, THz-wave–tissue interactions, THz dosimetry, THz exposures of biological molecules, cells, and tissues, thermal and nonthermal effects of THz waves

## Abstract

**Significance:** An increasing interest in the area of biological effects at exposure of tissues and cells to the terahertz (THz) radiation is driven by a rapid progress in THz biophotonics, observed during the past decades. Despite the attractiveness of THz technology for medical diagnosis and therapy, there is still quite limited knowledge about safe limits of THz exposure. Different modes of THz exposure of tissues and cells, including continuous-wave versus pulsed radiation, various powers, and number and duration of exposure cycles, ought to be systematically studied.

**Aim:** We provide an overview of recent research results in the area of biological effects at exposure of tissues and cells to THz waves.

**Approach:** We start with a brief overview of general features of the THz-wave–tissue interactions, as well as modern THz emitters, with an emphasis on those that are reliable for studying the biological effects of THz waves. Then, we consider three levels of biological system organization, at which the exposure effects are considered: (i) solutions of biological molecules; (ii) cultures of cells, individual cells, and cell structures; and (iii) entire organs or organisms; special attention is devoted to the cellular level. We distinguish thermal and nonthermal mechanisms of THz-wave–cell interactions and discuss a problem of adequate estimation of the THz biological effects’ specificity. The problem of experimental data reproducibility, caused by rareness of the THz experimental setups and an absence of unitary protocols, is also considered.

**Results:** The summarized data demonstrate the current stage of the research activity and knowledge about the THz exposure on living objects.

**Conclusions:** This review helps the biomedical optics community to summarize up-to-date knowledge in the area of cell exposure to THz radiation, and paves the ways for the development of THz safety standards and THz therapeutic applications.

## 1 Introduction

**Fig. 1:**
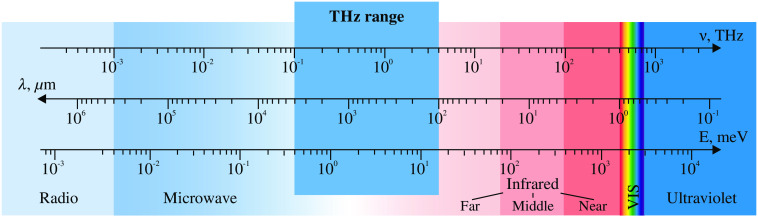
The THz range of the electromagnetic spectrum. Courtesy of the authors.

An increasing interest in biomedical applications of terahertz (THz) radiation, featuring the frequencies of 0.1 to 3.0 THz, the free-space wavelengths of ≃3  mm to 100  μm, or the quantum energies of ≃0.3 to 10 meV (see Fig. 1), has been observed during the past few decades,^1^^–^^5^ driven by rapid progress in THz technology.^6^^,^^7^ Numerous research papers demonstrate the potential of THz technology in label-free early noninvasive, least-invasive, and intraoperative diagnosis of malignant and benign neoplasms with different nosologies and localizations,^3^^,^^4^^,^^8^^–^^18^ sensing of glycated tissues and blood in the context of diabetes diagnosis,^1^^,^^19^^–^^21^ determining the degree of traumatic injuries,^22^^–^^25^ hydration levels,^26^^–^^29^ and viability^30^ of tissues. Along with the diagnostic applications, THz technology holds strong potential in therapeutics,^31^^,^^32^ for example, in nonthermal regulation in expression of genes associated with cancer and inflammatory diseases.^33^ Nevertheless, THz therapy is still at the initial stage of its development.

**Fig. 2:**
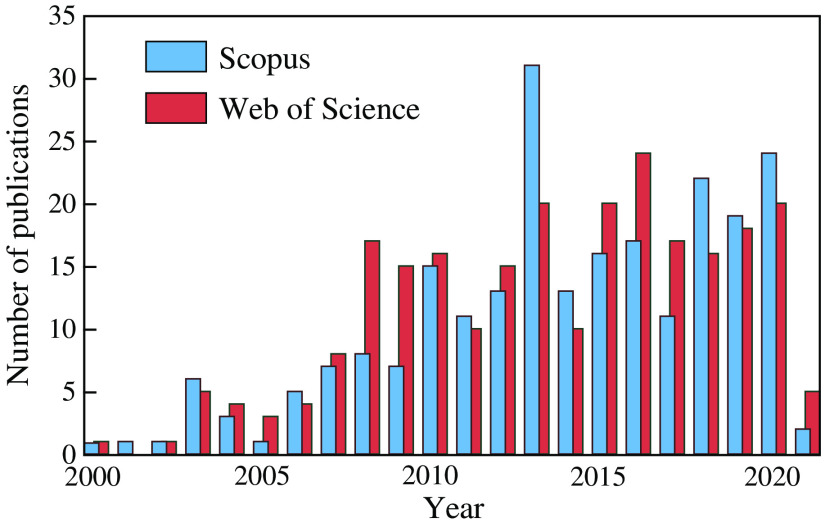
Increasing number of research items in the area of biological objects exposure to THz waves according to Scopus and Web of Science; report dated February 2, 2021. Courtesy of the authors.

Despite the attractiveness of THz technology for medical diagnostics and therapy, currently, the accumulated data on the safe limits of its effects on tissues and its harmlessness to the human body are rather limited.^34^^–^^37^ The existing recommendations of THz-radiation safety limits were developed relying on extrapolation of the data from the neighboring millimeter-wave (MMW) and infrared (IR) ranges.^38^^–^^42^ Therefore, THz-wave biological effects require thorough investigation for defining the safety limits in THz biomedical diagnostics and determining the optimal dose for THz therapeutics. In Fig. 2, an annually increasing number of research items according to Scopus and Web of Science is shown, illustrating a growing interest in the considered issues. To objectively uncover benefits and weaknesses of THz medical diagnosis and therapy and to define safe limits of tissue exposure to THz waves, appropriate modes of continuous-wave (CW) and pulsed THz exposure (average power, duration, and number of exposure cycles) ought to be systematically studied and analyzed.

In this review, we begin with the technical aspects of the effect of THz waves on biological objects. Next, we discuss and compare modern THz emitters and their applicability for studying biological effects. We then describe the three levels of organization of biological systems, at which the effects of exposure to radiation are usually studied:

• biological molecules,
• cultures of cells, individual cells, and cell structures,
• entire organs or organisms.

We perform an in-depth analysis of cell exposure to CW and pulsed THz radiation, considering different types of cells (such as blood, skin, neuronal, epithelial, and stem cells) and distinguishing two distinct mechanisms of the THz-wave–cell interactions:

• heating due to the strong THz-wave absorption by polar water molecules;^34^
• nonthermal effects, including changes in the deoxyribonucleic acid (DNA) molecule dynamics (local breaks of hydrogen bonds and the DNA chains’ melting) and gene expression.^43^^–^^45^

Original contributions of the authors to the described research areas are discussed, including studies of THz biological effects on neurons and fibroblasts, and evaluation of biological objects’ heating by pulsed and CW THz radiation. Finally, this review addresses the problem of adequate estimation of the THz biological effects’ specificity, as well as the problem of reproducibility of experimental data, originating from both rareness and uniqueness of the THz systems and absence of unitary irradiation protocols, which sometimes leads to contradictions of the results obtained by different research groups.^35^ Thereby, this review summarizes up-to-date knowledge in the area of cell exposure to THz radiation and poses important problems that hinder further developments in THz safety standards and in diagnostic and therapeutic applications.

## 2 THz-Wave--Tissue Interactions

Before proceeding to biological effects of THz waves, we should briefly overview some general principles of THz-wave–tissue interactions, which together with the rapid development of THz components and instruments^6^^,^^7^ cause significant interest to different THz applications.^2^^,^^34^^,^^46^^,^^47^ The following remarkable features of these interactions attract special attention:

• THz radiation is nonionizing in nature due to quite low photon energy, as compared with the ionization energy, which leads to dissociation of atoms and molecules.
• THz radiation interacts with free charges, low-frequency molecular motions, and collective excitations of media. Energy of THz quanta corresponds to the energy of hydrogen bounds and Van der Waals intramolecular interactions.
• Solid state materials and molecular crystals might be characterized by unique “fingerptints” in the THz range, i.e., resonant spectral absorption peaks.
• THz radiation is strongly absorbed by polar molecules, such as water in liquid and gas states. On the one hand, this makes THz waves very sensitive to the content and state (free or bound) of water in the measured object, including different biological tissues. On the other hand, this limits the depth of THz-wave penetration in biological tissues by only hundred or even tens of microns, depending on the frequency and tissue type.
• THz waves penetrate into various nonhydrated dielectric materials, such as plastic, paper, cloths, and wood, especially at the sub-THz frequencies.
• Structural inhomogeneities of many objects, including different biological tissues, are small at the THz-wavelength scale. This reduces the Mie scattering effects, increases the THz-wave penetration depth in such objects as compared to the visible and IR waves, and allows one to apply the effective medium theory for analysis and description.

**Fig. 3:**
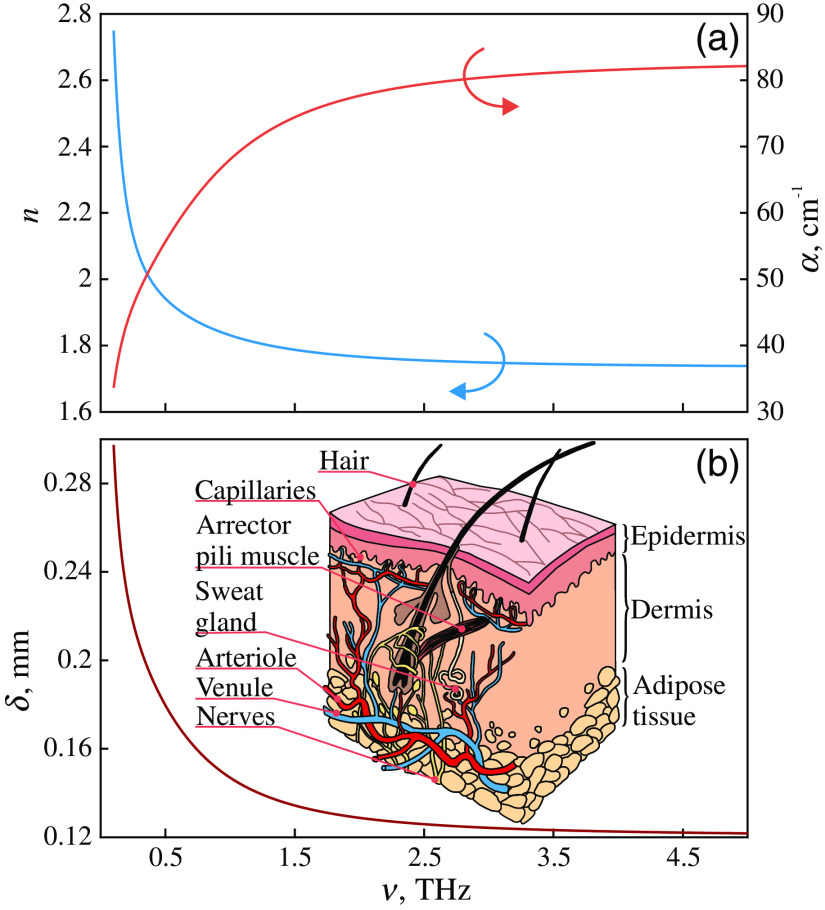
THz optical properties of the skin (epidermis). (a) Refractive index n and absorption coefficient α (by field), calculated from the double-Debye model described in Ref. 48. (b) THz-wave penetration depth δ (by field). Inset in (b) shows a scheme of the skin, where (in most cases) only the epidermis is probed by the THz radiation. Courtesy of the authors.

High water content is a general feature of all living organisms, which is of crucial importance for their interaction with THz waves. Indeed, water constitutes up to ≃60% (by weight) of the adult human body; and its content is ≃73% in tissue of the brain and heart, ≃83% in the lungs, ≃64% in the skin, ≃79% in the muscle tissues and kidney, and ≃31% in the bones. Blood constitutes 7% to 8% of the human body, which is as much as 4.5 to 6.0 l for adults. In Fig. 3, structure and effective optical properties of the skin are illustrated in form of the frequency-dependent refractive index n, absorption coefficient α (by field), and penetration depth δ=1/α (by field). The given data are calculated based on the double-Debye model of the tissues dielectric response at THz frequencies, introduced in Ref. 48. As it is shown in Fig. 3(b), the penetration depth δ decreases with increasing frequency ν, whereas it is smaller than 0.3 mm in the considered spectral range.

The above-mentioned features of the THz waves open wide capabilities of their use in different branches of biology and medicine, which are discussed later with an emphasis on THz exposure effects.

### 2.1 Dimensions of Tissue Components Versus the THz Wavelengths

Depending on the ratio between the dimensions of tissue structural elements δ and the free-space electromagnetic wavelength λ, one can expect distinct regimes of the electromagnetic wave–tissue interactions, which are governed by different physical regularities.^49^ When the tissue components are small in the wavelength scale (δ≪λ), the Rayleigh scattering regime takes place and tissues are assumed to be homogeneous. In such case, one can use the effective medium theory^2^ for describing the electromagnetic-wave–tissue interactions, which is widely applied in the MMW range, that bounds the THz gap from its low-frequency side.^50^ Otherwise, when the tissue structural elements have mesoscale dimensions (δ∼λ), or even appear to be large (δ≫λ), the Mie scattering effects occur and the radiation transfer theory should be applied to describe the interactions. This theory is widely used in the ultraviolet, visible, and IR bands;^51^ the latter is adjacent to the THz range at its high-frequency side.

**Fig. 4:**
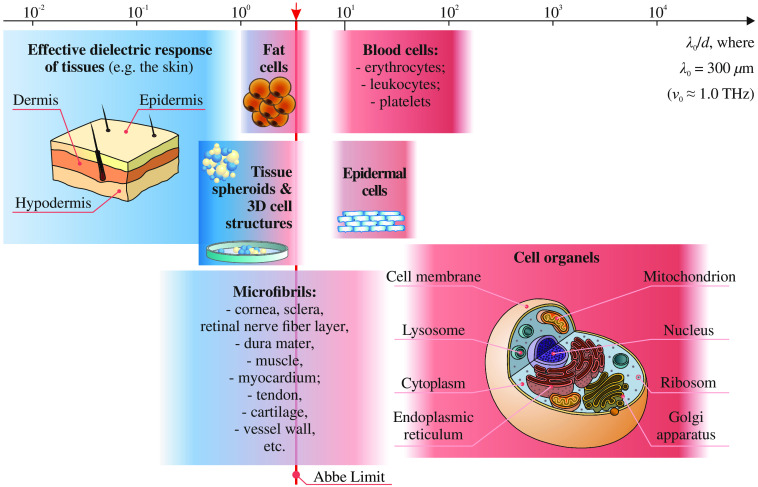
Dimensions of the tissue structural elements at the THz-wavelength scale. Typical dimensions of the tissue structural elements δ are normalized by the particular wavelength of $\lambda_{0}=300\;\mu m$ ($\nu_{0}≃1.0\;\mathrm{THz}$). Vertical solid red line defines the λ/2-Abbe diffraction limit. Courtesy of the authors.

In Fig. 4, dimensions of the typical tissue structural elements, such as microfibrils, separate cells, and cell organelles,^52^ are compared with the characteristic free-space THz wavelength of λ=300  μm (ν≃1.0  THz). Here, a vertical red solid line points out the ≃λ/2 Abbe diffraction limit of spatial resolution of lens- or mirror-based optical systems. On the one side, the majority of the tissue structural components are much smaller as compared with the defined THz wavelength, which allows using the effective medium theory for describing the THz-wave interaction with tissues comprised of such components.^2^ On the other side, numerous structural components of tissues are characterized by dimensions that are comparable to the THz wavelengths and, thus, become a source of the Mie scattering.

Despite the effective medium theory is more usual in THz biophotonics,^2^^,^^3^ an intermediate position of the THz waves between the IR waves (with their strong Mie scattering in tissues) and the MMW waves (almost insensitive to the tissue structural inhomogeneities) poses a challenging problem of selecting appropriate models of the THz-wave–tissue interactions.

### 2.2 Effective Medium Theory in the THz Range

The effective medium theory assumes tissues to be homogeneous at the THz-wavelength scale and describes the THz-wave–tissue interactions using models of their effective dielectric response.^2^^,^^3^ Such models define simultaneously frequency-dependent real $\varepsilon^{\prime }$ and imaginary $\varepsilon^{\prime \prime }$ parts of a complex dielectric permittivity

$$\overset{˜}{\varepsilon }=\varepsilon^{\prime }-i\varepsilon^{\prime \prime },$$ (1)

or real $n^{\prime }$ and imaginary $n^{\prime \prime }$ parts of a complex refractive index

$$\overset{˜}{n}=n^{\prime }-in^{\prime \prime }\equiv n-i\frac{c}{2\pi \nu }\alpha \equiv \sqrt{\overset{˜}{\varepsilon }},$$ (2)

where $c≃3\times 10^{8}\;m/s$ is the speed of light in free space, and α is an absorption coefficient (by field) in ${\mathrm{cm}}^{-1}$.

THz waves strongly interact with polar water molecules in liquid water (either free or bound), aqueous solutions, and tissue water.^2^^,^^3^^,^^53^ Therefore, content and state of water play dominant roles in the formation of the THz dielectric response of hydrated tissues.^21^ Similarly to the THz dielectric response of liquid water, aqueous solutions, and biological liquids,^2^^,^^3^ the THz-wave losses in tissues, which are defined by $\varepsilon^{\prime \prime }$ or α, possess no resonant features. In the THz range, complex dielectric permittivity of water and hydrated media is usually described by the relaxation models, among which the double-Debye model should be emphasised as the most widely applied in THz biophotonics^48^^,^^54^^–^^60^

$$\overset{˜}{\varepsilon }=\varepsilon_{\infty }+\frac{\Delta \varepsilon_{1}}{1+i\omega \tau_{1}}+\frac{\Delta \varepsilon_{2}}{1+i\omega \tau_{2}},$$ (3)

where ω=2πν is a circular frequency, $\tau_{1}$, $\tau_{2}$ and $\Delta \varepsilon_{1}$, $\Delta \varepsilon_{2}$ are times and amplitudes of the “slow” and “fast” relaxations, $\varepsilon_{\infty }$ is a constant dielectric permittivity at high frequencies [$\omega \gg {\left(2\pi \tau_{i}\right)}^{-1}$]. In Refs. 2 and 3, one can find parameters of the double-Debye model that are summarized for liquid water, as well as for different healthy and pathologically altered tissues *ex vivo* and *in vivo*.

**Fig. 5:**
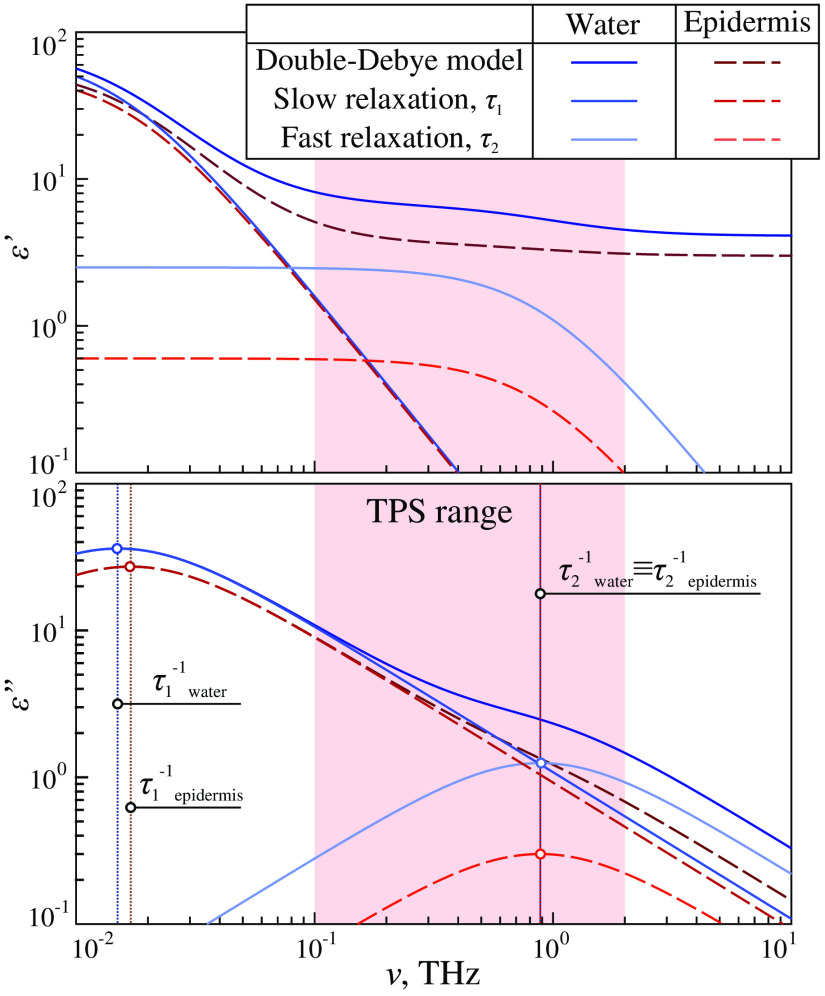
Double-Debye models of the complex dielectric permittivity $\overset{˜}{\varepsilon }$ for water and epidermis of the skin *ex vivo* reported in Ref. 48. Peach-colored area defines the spectral operation range of the THz-pulsed spectrometer that was used in Ref. 48 to measure the dielectric data. Courtesy of the authors.

In Figs. 5(a) and 5(b), real $\varepsilon^{\prime }$ and imaginary $\varepsilon^{\prime \prime }$ parts of the complex dielectric permittivity are plotted for the liquid water and epidermis of the skin *ex vivo* based on the double-Debye model parameters from Ref. 48, respectively, for liquid water: $\varepsilon_{\infty }=4.1$, $\Delta \varepsilon_{1}=72.2$, $\Delta \varepsilon_{2}=2.5$, $\tau_{1}=10.6\;\mathrm{ps}$, and $\tau_{2}=0.18\;\mathrm{ps}$; for epidermis: $\varepsilon_{\infty }=3.0$, $\Delta \varepsilon_{1}=56.4$, $\Delta \varepsilon_{2}=0.6$, $\tau_{1}=10.0\;\mathrm{ps}$, and $\tau_{2}=0.2\;\mathrm{ps}$. For both water and epidermis, $\varepsilon^{\prime \prime }$-curve is formed by the two broad absorption peaks, where one of them is attributed to the “slow” Debye relaxation $\Delta \varepsilon_{1}$ and centered far below the THz range [at the inverse relaxation time ${\left(2\pi \tau_{1}\right)}^{-1}$], whereas another one represents the “fast” Debye relaxation $\Delta \varepsilon_{2}$ and centered at the high-frequency edge of the THz range [at the frequency of ${\left(2\pi \tau_{2}\right)}^{-1}$]. In turn, $\varepsilon^{\prime }$-curve decays with increasing frequency, with some pronounced changes near the inverse relaxation times ${\left(2\pi \tau_{1}\right)}^{-1}$ and ${\left(2\pi \tau_{2}\right)}^{-1}$.

In Eq. (3), for free bulk water, the “slow” Debye relaxation $\Delta \varepsilon_{1}$ describes cooperative reorganization of water molecules connected by hydrogen bonds, whereas the “fast” Debye relaxation $\Delta \varepsilon_{2}$ represents vibrational motions of water molecules that are free from hydrogen bounds.^61^ Hydration of biological molecules in aqueous solutions and tissue might lead to more complex dielectric response.^62^^,^^63^ Electric charges of biological molecules create the electric field, which orients surrounding water molecules and leads to formation of hydrated water layers (shells) ^64^ with their unique relaxation dynamics. Such hydration can either change the parameter of the double-Debye model^65^ or lead to appearance of additional relaxation or quasiresonant terms in the dielectric permittivity model.^66^ Furthermore, ionic conductivity and spectral fingerprints of hydrated ions might also contribute to the THz dielectric response of biological systems.^67^^–^^69^ However, an in-depth review of modern research works in the area of the THz dielectric spectroscopy of pure bulk water, aqueous solutions of biomolecules, segregated water, and tissues is far beyond the scope of the present review, but it can be found in Refs. 2 and 21.

Despite the fact that the discussed double-Debye model is generally accepted in THz biophotonics, physically it is not rigorous. Indeed, on the one hand, it implies fitting of the experimental data by the two broad absorption bands, centered either beyond or at the edges of the THz range (see Fig. 5). In other words, when using the double-Debye model, one deals with something similar to the experimental data extrapolation. However, in the case of taking appropriate initial conditions during the fitting procedure, the double-Debye model yields very convenient parametrization of the spectral curves by only five independent parameters: $\varepsilon_{\infty }$, $\Delta \varepsilon_{1}$, $\Delta \varepsilon_{2}$, $\tau_{1}$, and $\tau_{2}$. On the other hand, the Debye kernels in Eq. (3) do not fulfill the sum rule for the oscillator strength and, thus, predict infinite number of charge carriers/dipoles N underlying the dielectric response of a medium:^70^

$$N\propto \int_{0}^{\infty }\omega \varepsilon^{\prime \prime }d\omega =\text{finite}.$$ (4)

To mitigate this drawback, the double-overdamped oscillator model was applied to describe the THz dielectric response of tissues in Ref. 15. As compared with the double-Debye model, a pair of the overdamped oscillators provides almost equal dielectric curves $\varepsilon^{\prime }$ and $\varepsilon^{\prime \prime }$ in the analyzed THz range but predicts considerably lower losses $\varepsilon^{\prime \prime }$ at high frequencies $\omega \gg {\left(2\pi \tau_{i}\right)}^{-1}$ and, thus, satisfies the sum rule. Other semiempirical relaxation models of a complex dielectric permittivity exist and are sometimes applied for fitting the THz data, including the Cole–Cole,^71^^,^^72^ Davison–Cole,^73^ or Havriliak–Negami ^74^ models. They imply more parameters to take into account possible asymmetry of the discussed broad absorption peaks in the $\varepsilon^{\prime \prime }$-curves.

Moreover, the aforementioned models of the tissue complex dielectric permittivity in the THz range have quite qualitative character. In fact, earlier, the two broad absorption peaks, predicted by these models, were observed experimentally mostly for the liquid water and aqueous solutions;^53^ at the same time, for biological tissues, they have not been observed experimentally yet involving broadband dielectric measurements, which is, probably, due to complexity of such experiment. Thus, these relaxation models are applied to describe the tissues response only intuitively. Moreover, structure, relaxation dynamics, and THz response of liquid water and, thus, water-containing biological systems can be more complex. Their dielectric permittivity model can comprise three or even more relaxation terms, each representing a particular fraction of free/bound or segregated water, with distinct electrodynamic characteristics, that can be predicted, for example, by numerical simulations using first principles.^66^^,^^75^ Detailed overview of picosecond dynamics of water and water-containing media, as well as their THz dielectric response, deserves separate in-depth review, being out of the scope of this paper.

Nevertheless, information about a tissue complex dielectric permittivity (or a complex refractive index) helps to model the THz-wave–tissue interaction within the framework of classical electrodynamics, which is of crucial importance in THz spectroscopy and imaging of tissues aimed at medical diagnosis of different pathological processes. These data can be also useful in THz exposure of tissues since they provide an information about the penetration depth of THz waves into the tissues δ=1/α or the volume of the exposed tissue $V≃\pi D^{2}\delta /4$, where D is the THz beam spot diameter.

### 2.3 Fingerprints of the Mie Scattering in the THz Range

Despite the aforementioned effective medium theory is widely applied in THz biophotonics, a lot of biological objects and tissues possess structural inhomogeneities with the dimensions comparable to the THz wavelengths; see Fig. 4. For such objects, the Mie scattering effects should be taken into account.^51^

For example, in Refs. 3 and 76, considerable structural inhomogeneities of fibrous connective tissues of the breast were studied both experimentally, using the 0.15λ-resolution THz solid immersion microscopy,^77^ and theoretically, using analytical methods of the Mie scattering theory. THz optical properties of separate fat cells and their agglomerates were found to differ from that of the surrounding fibrous connective tissues of the human breast *ex vivo*. Such cells form sources of the Mie scattering at THz frequencies. The calculated parameters of such scatterers (i.e., their scattering phase function, differential and integral cross sections, and scattering anistoropy factor) considerably differ from those inherent to the Rayleigh scattering regime. In Refs. 17 and 29, THz solid immersion microscopy revealed structural heterogeneities of intact brain tissues and glioma model 101.8 from rats *ex vivo*, as well as heterogeneous character of decellularized bovine pericardium collagen matrices. Finally, in Refs. 78–80, polarization-sensitive THz imaging provided useful information for the differentiation between healthy and pathologically altered tissues, which also indirectly justified the Mie-like character of the THz-wave scattering in tissues. Namely, in this imaging modality, one observes changes in polarization of the THz wave interacting with tissues, which could not be described in the framework of the above-mentioned effective medium theory. All these experimental findings stimulate further research and development of approaches for describing the THz-wave–tissue interactions that simultaneously account for the dispersion and absorption properties of tissue components, as well as geometry and organization of scatterers in tissues.

This approach could rely on the radiation transfer theory, applied for describing the electromagnetic-wave–tissue interactions in the ultraviolet, visible, and IR ranges.^51^ The basis of the radiation transfer theory is a radiation transfer equation,^49^ which defines a radiance $I(r,\hat{s})$ at a point r in the direction $\hat{s}$:

∂I(r,s^)∂s=−μtI(r,s^)+μs∫4πI(r,s^′)p(s^,s^′)dΩ′+I0(r,s^), (5)

where $\mu_{s}$ and $\mu_{t}$ are the scattering and total (power absorption $\alpha_{p}$ and scattering $\mu_{s}$) coefficients in ${\mathrm{cm}}^{-1}$, p(s^,s^′) is the scattering phase function, $\Omega^{\prime }$ is the unit solid angle around s^′, and $I_{0}(r,\hat{s})$ is the radiance of internal sources. Scattering phase function accounts for the characteristic angular distribution of the Mie scattering, including anisotropy of the tissue scattering properties.

Studying the scattering effects in tissues seems to be important for the THz diagnosis, where they can either complicate differentiation of tissues or become a source of additional useful information. In THz exposure of tissues, scattering effects can play an important role due to the effects of electromagnetic field confinements at the shadow side of mesoscale dielectric obstacles that can considerably (by few orders of magnitude) enhance local irradiance of tissues by THz waves and, thus, increase efficiency of tissue exposure to THz waves.

## 3 Modern THz-Wave Emitters

Currently, tremendous efforts are still paid to boost the development of THz emitters. The THz spectral range and, particularly, the exposure technologies attract increasing interest owing to the appearance of solutions for THz-wave generation.^6^^,^^7^ Such emitters working either in CW or pulsed mode allow generation of the output power ranging from the nanowatts to kilowatts. Here, we briefly overview the recent progress in this area with an emphasis on the practically important devices that are capable of solving modern research and engineering problems of tissue exposure to THz waves.

Despite a wide variety of existing THz-wave emitters with their rich diversity of operation principles and regimes, output frequencies, and powers, this review focuses only on widespread systems that went beyond the research work of a physical laboratory and, in our opinion, can be applied for studying biological effects of THz waves or even for therapeutic applications of THz technology. Thus, in Tables 1 and 2, we summarize only the typical technical characteristics of the discussed principles and devices. However, we should stress that unique THz systems developed for experiments in a physical laboratory can provide somewhat higher performance, i.e., broader spectral range, higher power, and wider tunability. In fact, in Tables 1 and 2, we deliberately underestimate the spectral operation ranges of some THz emitters. We consider only those parts of the spectral operation ranges, where THz emitters provide reliable output power, and neglect, at the same time, much less-intense side lobes and tails of the emitted THz spectra.

**Table 1:** Typical CW THz emitters.

Source and principle	Frequency	Output power	Tunable bandwidth	Conversion efficiency	Operation temp.	Refs.
Interaction between electron beam and back-traveling electromagnetic wave	<1.0 THz	mW to kW	++	$<10^{-2}$	293 K	82–86
DFG and PG						
Nonlinear optical process by frequency difference of two optical beams or two stimulated photons in a nonlinear crystal	<5.0 THz	mW to kW	++++	$<10^{-3}$	293 K	6,88–96
OPGL						
Rotational transitions between vibrational modes in optically pumped molecular gases	<8.0 THz	μW	++	$<10^{-2}$	293 K	97–102
Diode-based frequency multiplier						
Frequency multiplication of diode-based microwave sources with nonlinear media	<2.5 THz	μW	+++	<0.4	< 293 K	103–109
Photomixing						
Photocurrent oscillation in photomixer by frequency difference of two optical beams	<4.0 THz	μW to mW	++++	$∼10^{-6}$ to $10^{-3}$	293 K	110–114
Quantum cascade laser						
Electrons cascading in intersubband transitions with quantum well semiconductor heterostructures	1.0 to 6.0 THz	mW to W	++++	<0.3	<200 K	115–121
Gyrotron						
Coherent bremsstrahlung radiation between the gyrating beam electrons and electromagnetic wave	<1.5 THz	W to kW	++	$10^{-3}$ to $10^{-1}$	293 K	151–160

**Table 2:** Typical pulsed THz emitters.

Source and principle	Frequency	Average power	Conversion efficiency	Operation temp.	Refs.
Generation in PCA					
Acceleration and decay of free charge carriers in biased semiconductor gap pumped with ultrafast laser pulse	<3.0 THz	μW-mW	$∼10^{-6}-10^{-2}$	293 K	122–124,126,130–132
OR					
Time varying nonlinear polarization in nonlinear crystal pumped with ultrafast laser pulse	<7.0 THz	μW-mW	$∼10^{-7}-10^{-2}$	293 K	136–150
Gyrotron					
Coherent bremsstrahlung radiation between the gyrating beam electrons and electromagnetic wave	<1.5 THz	kW–MW	<0.6	293 K	161–164

### 3.1 CW or Quasi-CW THz Sources

#### 3.1.1 Backward wave oscillator

In terms of the CW sources, backward wave oscillator (BWO)^81^ is one of the well-known methods to generate intense THz radiation. Nanosecond pulse with a maximum output power in kW level is also achievable by this method.^82^ In BWO, the THz radiation is generated based on the interaction between an electron beam and an electromagnetic wave traveling in the opposite direction. An electron beam is emitted from a heated cathode and collimated by magnetic field. A modulation device, such as metal grating or corrugated rectangular waveguide, is carefully arranged to modulate the electron beam and give rise to a surface electromagnetic wave. The frequency of the electromagnetic wave could be tuned by adjusting the velocity of electron beam, thus, obtaining the THz radiation.^83^ Mineo et al. demonstrated a BWO featuring a 20% tuning bandwidth at the central frequency of 1.0 THz with the output power of ∼100  mW.^84^ In 2015, a plasma wave-assisted BWO was employed to radiate THz waves in the range of 186 to 202 GHz with the maximum power of 20 W by adopting a pseudospark-sourced electron beam.^85^ Recently, oversized BWOs operated in the frequency ranges of 260 to 340 GHz and 310 to 390 GHz were studied theoretically and experimentally. Two BWO types were designed and tested, and the detected peak power of a few hundred of mW was obtained by adopting the optimal structures.^86^

BWOs are capable of operation in a pure CW regime^81^ with the monochromatic spectral output, quite wide spectral tunability, the spectral line-width of down to $∼10^{-5}\;\nu$, and the average output power of $∼10^{-1}$ to $10^{-4}\;W$. Note that the output power decreases with increasing operation frequency ν. Such CW BWOs are widely applied in the THz dielectric measurements.^81^

#### 3.1.2 Difference frequency generation and parametric generation

The principles of difference frequency generation (DFG) and parametric generation (PG) are all based on nonlinear optical process.^6^

For DFG, two optical beams featuring narrow bandwidth and slightly different frequencies colinearly propagate into a nonlinear crystal, leading to a THz radiation generated by the second-order nonlinear polarization at the difference frequency of the two optical beams.^87^ So far, various nonlinear crystals, for example, GaSe^88^ and GaP,^89^ were utilized to generate radiation up to 5.0 THz based on DFG. The output THz power of DFG was also improved from the mW to kW level. In 2014, by stacking four GaP plates reversely, the highest output power of 2.73 kW was achieved at around 2.7 THz and the conversion efficiency was demonstrated to be $∼10^{-3}$. It was also proved that the reversed GaP plates demonstrate stronger photon conversion than aligned GaP plates.^90^

For PG, only one optical pump beam is used to stimulate two photons, one is commonly named as “idler,” whereas another is the THz one.^91^ The THz wave could obtain dramatic amplification when a phase-matching condition is satisfied. The tuning of the THz frequency is continuously achievable by controlling the relative angle between the idler and pump beam.^92^^,^^93^ Kawase et al. developed a widely tunable THz wave parametric generator in the range of 0.7 to 2.4 THz using injection seeding. The maximum peak output THz power exceeded 200 mW.^94^ The performance of injection-seeding-based parametric generator was further improved through utilizing the laser light scattering from a nonlinear crystal by the same group. It helped to achieve the THz radiation in the range between 0.7 and 3.0 THz and improve the maximum output power from the mW to kW level.^91^ Afterward, the maximum output power was increased to ≃50  kW by Minamide et al.^95^ and Hayashi et al.,^96^ respectively, with or without using the microchip laser.

#### 3.1.3 Optically pumped gas laser

Optically pumped gas laser (OPGL) features the capability of generating THz waves with narrow linewidth.^6^ The basic principle of OPGL is based on population inversion in the gas molecules. This population inversion occurs when the gas molecules, possessing lower vibrational mode, are optically pumped. Since the gas molecules feature permanent dipole moments, the induced rotational transitions between high and low vibrational modes can be directly coupled to electromagnetic radiation, thus, generating THz wave.^97^

Various molecular gases have been demonstrated for generating THz wave in the range of 0.1 to 8 THz, such as ${\mathrm{CH}}_{3}F$, ${\mathrm{CH}}_{3}\mathrm{OH}$, ${\mathrm{NH}}_{3}$, and ${\mathrm{CH}}_{2}F_{2}$.^98^^,^^99^ Recently, Chevalier et al. proposed a research on widely tunable THz gas laser, in which over 37 laser lines were observed from 0.25 to 0.96 THz using optically pumped nitrous oxide ($N_{2}O$) laser. The possibility of achieving electromagnetic waves with frequencies above 1.0 THz with a mW-level output power based on QCL pumped gas laser was predicted by theoretical analysis.^100^ Wienold et al.^101^ presented a gas laser based on QCL pumped NH153 molecules; several laser lines with the output power of up to 30  μW were obtained around 4.5 THz by exploiting the molecular symmetry and employing an alternative resonator. Liu et al.^102^ proposed a tunable optically pumped ${\mathrm{CH}}_{3}F$ gas laser with a germanium ealton serving as spectrum splitter, four THz laser lines corresponding to a conversion efficiency of $∼10^{-3}$ were observed in the range of 0.5 to 1.7 THz by tuning the incident angles and pump wavelengths.

#### 3.1.4 Diode-based frequency multiplier

Diode-based frequency multiplier is a solid-state THz source, which implements microwave multiplication from IMPATT (IMPact ionization Avalanche Transit-Time), Gunn, tunnel, and other diodes. By making appropriate operations, the negative resistance devices could be built based on the aforementioned diodes. A resonator coupled with the negative resistance devices is used to generate an AC signal intended for CW microwave radiation. In this process, the zero-attenuation oscillator is a crucial element for the microwave radiation, which is obtained through adjusting the magnitude of the negative resistance devices. Consequently, the THz wave can be received after the microwave passing through a series of frequency multiplier chains, where the Schottky diodes are commonly used as a nonlinear media for harmonic generation of microwave.^6^

The diode-based THz sources are usually compact and operate at room temperatures, which is convenient for practical application. The average output power reaches the mW level and can be improved by cooling. For example, in 2002, Maiwald et al.^103^ demonstrated a peak output power of 120  μW (at 1.178 THz) at room temperatures and an improved peak output power of 190  μW (at 1.183 THz) by cooling down to 113 K. Most of the reported multipliers feature the output power of 1 to 200  μW at the frequencies of <2.5  THz.^104^^–^^109^

#### 3.1.5 Photomixing

Photomixing, also known as optical heterodyne generation, is one of the solid-state CW THz generation technique, capable of making narrow band signals with exceptional tunability.^110^ Two CW laser beams with identical polarization and slightly different frequencies are utilized to generate a THz beat, which illuminates a photomixer, resulting in a modulated photocurrent. The induced photocurrent oscillations adjusted by tuning the frequency difference between the applied lasers determine the desired parameters of THz radiation.^111^ The main well-known disadvantage of this technique is the relatively low output power due to the requirement of CW optical excitation and shortage of semiconductor materials with high thermal conductivities.

Low-temperature-grown GaAs has been employed as the photomixer material in the last few years to generate THz radiation with the frequencies up to 3.0 THz.^112^ The typical optical-to-THz conversion efficiency for the photomixing method is $∼10^{-6}$ to $10^{-5}$, which limits the output power in the order of mWs.^113^ Despite tremendous efforts have been put in the enhancement of photomixer performance, the output CW power is still limited by the mW level. Recently, Ironside et al.^114^ proposed a metamaterial-enhanced photomixer by employing a metal–semiconductor–metal cavity; such photomixer is characterized by the conversion efficiency of $∼10^{-3}$ and is capable of generating THz radiation in the mW range.

#### 3.1.6 Quantum cascade laser

Quantum ascade laser (QCL) is undisputedly the only solid-state source that can generate THz radiation above 1.0 THz with an average output power at W level.^115^ The QCL realization is generally based on electrons cascading in intersubband transitions. Driven by electric field, the electrons are injected through a semiconductor heterostructure possessing multiple quantum wells, during which the electrons transit from a state with higher valence band energy to a lower state, thus, generating radiative emission. The efficient laser emission can be obtained, when a large population inversion condition is satisfied.^6^ The QCL tunability is mainly based on the structure of the semiconductor that offers superior bandwidth as compared with the diode-based sources.

To date, the high-power QCLs, operating in the frequency range of 1.0 to 6.0 THz, were demonstrated, with a few-W output power in pulsed mode and up to hundreds of mW in CW.^115^^–^^117^ In Ref. 118, Li et al. proposed a surface-plasmon waveguide embedded with an active region featuring a bound-to-continuum transition, based on which a THz QCL with the peak-pulsed output power of >1  W from the single facet at 3.4 THz was demonstrated. Williams et al.^119^ proposed CW QCL, with the power of 138 mW and the frequency of 4.4 THz, that employs a semiinsulating surface-plasmon waveguide and a resonant-phonon depopulation scheme. With 10 years of development, Wang et al.^120^ significantly improved the CW QCL output power to 230 mW by adopting a hybrid bound-to-continuum transition and resonant phonon extraction. One of the main challenges for QCL technology is the operation at room temperatures since the output power of QCLs decreases drastically as the temperature increases. The highest-reported operation temperature for QCLs without magnetic field assistance is close to 200 and 130 K for the pulsed and CW modes, correspondingly.^121^

### 3.2 Pulsed THz Sources

#### 3.2.1 Photoconductive antennas

The initial exploration of pulsed broadband THz wave generation using photoconductive antenna (PCA) could be traced back to 1970s to 1980s.^122^^–^^125^ The PCA generation principle is based on the free charge carries (electron and hole) excited in a biased semiconductor gap with femtosecond laser pulses.^126^^–^^128^ These free charge carries are accelerated by the applied static or alternating electric field that gives rise to a transient photocurrent. Simultaneously, the density of charge carries goes down due to the their trapping and recombination. The pulsed THz radiation is generated during the acceleration and trapping of these free charge carries. The decay time of the charge carries, which influence the duration and spectral bandwidth of thus generated THz pulse, is defined by the lifetime and mobility of carriers in a photoconductor. Therefore, photoconductors with shorter carrier lifetime are preferred to generate THz pulses with a broader frequency-domain bandwidth.^126^^,^^129^

To date, tremendous studies have been done to improve the performance of PCAs. Berry et al.^130^ demonstrated a plasmonic photoconductive THz source with enhanced output power by employing two-dimensional plasmonic contact electrodes. An average output power of ≃0.25  mW, which is 20 times higher than conventional PCAs, was achieved in the frequency range of up to 1.5 THz. In Ref. 131, Yang et al. reported a photoconductive emitter with the optical-to-THz power conversion efficiency as high as 7.5% using three-dimensional (3D) plasmonic contact electrodes. A broadband THz wave in the 0.1 to 2.0 THz range with the output power higher than 0.1 mW was experimentally observed at a 1.4-mW optical pump. Thereby, as compared with conventional PCA-based THz sources, the above-mentioned hybrid PCAs offer higher optical-to-THz conversion efficiency.^130^^,^^132^^–^^135^

#### 3.2.2 Optical rectification

Optical rectification (OR) is one of the most commonly used methods to generate pulsed THz radiation, which is based on second-order nonlinear optical effect. In the OR process, an ultrafast optical pulse is normally employed to illuminate a nonlinear optical crystal. The pulsed THz radiation is then generated by the time-varying nonlinear polarization.^136^ The bandwidth of the pulsed THz wave is roughly inversely proportional to the duration of the ultrafast optical pulse. The intensity of generated THz wave can be optimized by aligning the polarization of the incident optical beam.^6^

Schneider et al.^137^ and Venkatesh et al.^138^ demonstrated broadband THz radiations in the ranges of 0.4 to 6.7 THz and 0.1 to 3.0 THz, respectively, using a nonlinear organic salt. A near single-cycle THz pulses with the average power of 100  μW were demonstrated by the lithium niobite crystal (${\mathrm{LiNbO}}_{3}$) pumping with femtosecond optical laser pulses. The maximum energy of up to 10  μJ was detected at the frequency of 0.5 THz, which corresponds to the optical-to-THz energy conversion efficiency of $∼6\times 10^{-4}$.^139^ The maximum energy of pulsed THz wave radiated from ${\mathrm{LiNbO}}_{3}$ crystal was improved to 180  μJ by Jang et al.^140^ The highest average THz power of about 66 mW in the 0.1 to 4.0 THz range has been obtained with a few-cycle ultrafast laser-driven THz source by Meyer et al.^141^ through optimizing pump spot size and pump pulse duration. Both studies achieved a conversion efficiency on the order of $∼10^{-4}$. In Refs. 142 and 143, by satisfying the phase-matching condition, the conversion efficiency was dramatically improved to $∼10^{-2}$, pulsed THz generation with the pulse energies of 20 and 270  μJ and the frequency ranges of 0.1 to 5.0 THz and 0.1 to 6.0 THz was demonstrated.

In Ref. 144, Tripathi et al. demonstrated high-power THz-wave generation via OR of femtosecond pulses in 4-dimethylamino-N-methyl-4-stilbazolium tosylate crystal using a fiber-based laser pump, which paths the way for creating ergonomic high-power THz emitters. In Refs. 145–148, THz-wave generation was demonstrated in ≃0.1 to 10.0 THz range, using OR of near-IR femtosecond laser pulses (a Cr:forsterite laser system with a TW peak power) in a nonlinear organic crystal, with the output pulse duration and energy of ≃0.5 to 0.7 ps and <75  μJ, respectively. An experimental system for studying the THz-wave biological effects was developed relying on these unique THz setups.^149^^,^^150^

### 3.3 THz Gyrotrons

Gyrotron is one of the most powerful THz-wave sources in both CW and pulsed mode. Therefore, it is increasingly contributing to many application fields, such as nuclear magnetic resonance with signal enhancement using dynamic nuclear polarization, electron cyclotron resonance heating of fusion plasma, electron spin resonance spectroscopy, and x-ray detected magnetic resonance.^151^^,^^152^

The operation principle of gyrotron is based on the electron cyclotron resonance maser instability. The azimuthal and axial bunching of the helically moving beam electrons occurs due to the electromagnetic field stimulation, resulting in a proper synchronism between the electron beam and the electromagnetic wave excited in the resonant cavity, during which the transverse energy of the gyrating beam electrons can be transferred via coherent bremsstrahlung radiation for enhancing the excited electromagnetic wave.^153^ Due to the above principle, gyrotron generally operates at a fixed frequency close to the resonance frequency, and there are two ways that are commonly adopted for the frequency tunability in gyrotrons: tuning the magnetic field intensity or harmonic number of the cyclotron resonance. Nowadays, the highest frequency that can be achieved with the gyrotrons has already exceeded 1.0 THz for both CW and pulsed mode.

A CW gyrotron that is based on a 20-T superconducting pulsed magnet achieved for the first time a breakthrough of 1.0 THz.^154^ Its operation frequency and the output power were as high as 1.08 THz and ∼100 to 200 W, respectively. Nevertheless, majority of the reported CW gyrotrons possess an output power at W-level and sub-THz operation frequencies,^155^^–^^157^ whereas a few can obtain kW-level output power. For example, in Ref. 158, a continuously tunable CW gyrotron with the maximum output power of ∼400  W was demonstrated at the vicinity of ∼0.395  THz, with the corresponding maximum energy conversion efficiency of 4.3%. Another CW gyrotron, which was developed for the high frequency material processing, features the output power and operation frequency of ∼2  kW and 0.3 THz, respectively, operates in the magnetic field of up to 12 T, and provides the maximum power efficiency of ∼15%.^159^^,^^160^

For pulsed gyrotrons, the output power is even higher, which is generally at the level of kW and up to MW.^152^^,^^156^^,^^161^ For example, the MW-level gyrotrons with the output frequencies of 110 and 170 GHz were developed based on the depressed collector and the low-loss synthetic diamond window for efficiency improvement and high power Gaussian beam output.^162^ For these systems, the maximum efficiency of ∼57% was obtained at a 1.1-MW short pulse operation. The generation frequency of the pulsed gyrotron was significantly increased over 1.0 THz by the research group from IAP RAS through the use of intense magnetic fields. In Ref. 163, the radiation power and energy of ∼1.5  kW and 75 mJ, respectively, in a single shot regime were observed at the frequency of 1.022 THz with the magnetic field of ∼38.5  T. Afterward, a pulsed gyrotron was reported to operate at even higher frequency in the fundamental cyclotron resonance with a magnetic field strength of ∼50  T.^164^ The highest generation frequency of 1.3 THz was achieved, whereas the average output power was ∼0.5  kW.

## 4 Terahertz Exposure of Biological Objects with Different Levels of Organization

Rapid progress in THz technology, together with appearance of effective THz optoelectronic components and THz-wave generation principles, forces a translation of the THz technology into everyday practice, as well as increase an effect of THz radiation on humans. This rises the questions about the safety limits of biological systems’ exposure to THz waves. Defining the permissible exposure doses is impossible without a knowledge of the mechanisms of the THz-radiation–biological objects interactions during the THz exposure.^37^ Two distinct mechanisms of such interactions are usually distinguished.

### 4.1 Thermal Mechanism of THz Exposure

**Fig. 6:**
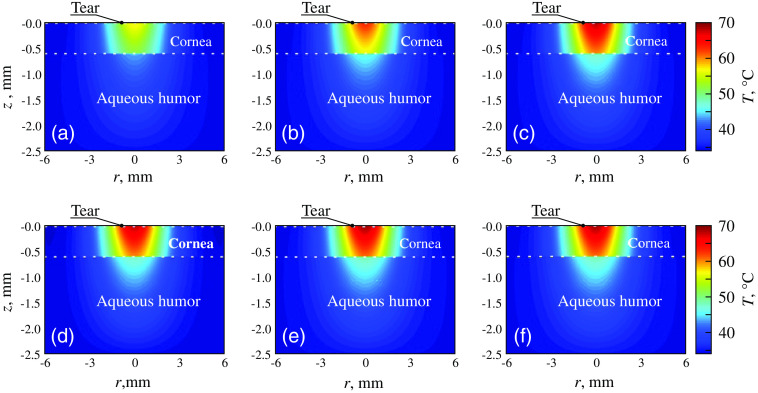
Simulated time-dependent evolution of the temperature distributions in cornea heated by the THz beam with the radius of 3 mm and the irradiance of $0.6\;W/{\mathrm{cm}}^{2}$, for the exposure times of (a) 10, (b) 30, (c) 60, (d) 180, (e) 300, and (f) 1200 s. Adapted from Ref. 169 with the permission of MDPI.

In the thermal mechanism, THz radiation causes heating of the irradiated object due to the strong THz-wave absorption by water.^34^^,^^41^^,^^165^^,^^166^ Such mechanism is predominantly observed when using CW THz emitters. Mathematical modeling of THz-radiation interactions with different biological tissues^167^^,^^168^ shows that even quite low duration of THz exposure (such as few milliseconds) can cause notable increase in the tissue temperature.^168^^,^^169^ An increase in the cornea temperature of up to 70°C was shown when it was exposed to a CW 1.0 THz radiation with the irradiance of $0.6\;W/{\mathrm{cm}}^{2}$; see Fig. 6. At the same time, experiments on THz exposure effects are often performed under thermostatically controlled conditions at physiological temperatures of ≃37°C, which prevents general heating of an exposed object.^42^^,^^170^^–^^172^

Several research groups reported the presence of microthermal effects of THz exposure that are difficult to detect during the experiments.^34^^,^^173^ However, significance of such effects still remains unexplored. The THz radiation is not perceived by the living systems as a thermal agent. This can be illustrated by special biological tests, such as studying the expression of genes associated with heat shock proteins. Their expression increases while the temperature rises by only several degrees from its optimal value. For example, the expression increases considerably at 41°C for humans.^34^^,^^171^

### 4.2 Nonthermal Mechanism of THz Exposure

To explain the nonthermal effects of THz exposure, theories of the Fröhlich long-range coherent oscillations,^174^ the Davydov molecular solitons,^175^ and the coherent acoustoelectric waves^176^ are commonly applied. Namely, Fröhlich postulated the presence of collective oscillations of electric dipoles in biological macromolecules and membranes,^173^^,^^177^ which can interact with low-energy quanta, including that from the THz range. These dipole coherent oscillations are due to the nonlinear interaction between modes, as well as to the energy flow into the lowest-frequency mode. Cell metabolism serves as a source of energy, whereas the nonlinearity is caused by a strong static electric field on the cell membranes.^178^ Modern experimental data provide confirmation of this hypothesis. For example, consider the first experimental observation of the Fröhlich condensation in a protein structure, reported few years ago in Ref. 179. Theoretical studies are carried out aimed at describing the Fröhlich condensates.^180^^–^^182^ This effect was analyzed in the semiclassical ^181^ and quantum frameworks,^182^ whereas the full-quantum statistical theory relies on the nonequilibrium Fröhlich condensate motion equations.

The theoretical calculations revealed that, under certain conditions, a resonant (linear or nonlinear) interaction between the THz wave and DNA is possible.^183^^,^^184^ Such an interaction significantly changes the molecular dynamics and leads to the local breaks of hydrogen bonds in DNA chains, which induces changes in gene expression. Based on a simple nonlinear model of DNA dynamics, it was demonstrated that THz radiation can influence both the vibrational excitations and proton motion in DNA hydrogen bonds. When the radiation frequency matches the vibrational mode, localized excitations in the form of dissipative solitons are generated.^185^ The low-amplitude collective breathing of the DNA modes can serve as precursors of generation of the transcription bubbles and other large-scale conformation changes.

Resonant interactions can be observed when using high-power pulsed THz sources.^43^^,^^186^^,^^187^ Indeed, while the average power of picosecond THz pulses is usually quite low (on the order of μW or mW), their peak powers can be as high as 1 to 30 GW.^145^^,^^146^^,^^188^ Such powerful THz waves can pass the cytoplasmic and nuclear membranes.^45^^,^^189^

**Fig. 7:**
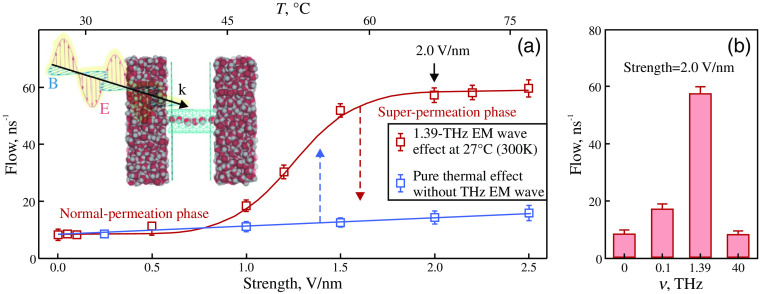
Numerical data on transition to a superpermeation phase of confined water across a one-dimensional (1D) water channel, that is, nonthermally modulated by THz radiation. (a) Water permeation is strongly and nonlinearly modulated by a 1.39-THz radiation (red squares), but weakly and linearly affected by temperature (blue squares). Inset shows a scheme of the simulated system, where red and gray balls indicate the oxygen and hydrogen atoms, respectively, whereas cyan tube stands for the 1D water channel, and two cyan sheets denote the supporting membrane. (b) Dependence of the permeation transition on the THz-wave frequency. The electric field strength of 2.0  V/nm is applied, corresponding to the region of the high plateau, at which the superpermeation phase occurs under the action of a 1.39-THz radiation. Adapted from Ref. 191 with the permission of the American Chemical Society.

Nonthermal effects of THz waves can occur for other structural components of living cell, such as proteins^179^ and membranes.^165^ An important role in THz-radiation–biological system interactions is played by water molecules, which are associated with biomolecules and impact their long-range interactions, collective vibrations, and transitions. Since THz radiation is strongly absorbed by water, the latter is involved into interaction with rapidly alternating electromagnetic fields.^32^^,^^190^ Molecular dynamics simulations revealed an enhanced permutation (by ≃1 order of magnitude) of confined water molecules across a water membrane channel, which is caused by a 1.39-THz radiation with a limited thermal effect; see Fig. 7. The underlying mechanism is due to a combination of the strength matching and frequency resonance between a relatively weak stimulus and the hydrogen bond network of confined water, rather than the bulk water outside. Such combination causes an anomalously structural phase transition of only the confined water while efficiently limiting the thermal effect of bulk water.^191^

### 4.3 Dosimetry in the THz Range

Currently, no comprehensive safety standards exist that consider both thermal and nonthermal THz-wave effects and regulate applications of the 0.1 to 10.0 THz radiation for general population and occupational exposure.^37^ On the one hand, exposure to radiation with the frequencies of <0.3  THz is governed by the International Commission on Non-Ionizing Radiation Protection (ICNIRP).^40^ It considers the frequency range of 100 KHz to 300 GHz and was updated in 2020.^192^ On the other hand, exposure to radiation with the frequencies of >0.3  THz is regulated by the ICNIRP standard 2013.^193^ This standard defines exposure to laser radiation with the 180 nm to 1 mm wavelengths, and it is based on the proven thermal effects. However, there is a significant discrepancy between these two guidelines, namely, the >0.3  THz standard allows for the 20 to 100 times higher exposure intensity, as compared with the <0.3  THz one.

For the exposure duration of >10  s and the frequencies >0.3  THz, the maximum power density is limited by $1\;\mathrm{kW}/m^{2}$. Whereas for the frequencies <0.3  THz, the general public exposure limit is two orders of magnitude lower, i.e., $10\;m^{2}$. Such a discrepancy between the two standards is due to different rules of the exposure threshold selection. The first standard^192^ defines the safety limits based on the increase in the body core temperature by only 1°C, i.e., from 37°C to 38°C. Such a small deviation of the body temperature can not cause any considerable hazard to an organism. In turn, the second standard^193^ is based on the 45°C temperature threshold of the skin injury. At the same time, both standards do not account the nonthermal effects of the THz waves.^194^ Meanwhile, the International Agency for Research on Cancer (IARC) of the World Health Organization (WHO) classified radiofrequency radiation in the 30 kHz to 300 GHz range as a human carcinogen, group 1.^195^

From a health risk perspective, ICNIRP are generally interested in the fraction of electromagnetic-wave power, which is absorbed by biological tissues and leads to their heating. This is typically described as a function of a relevant dosimetric quantity.^40^^,^^192^ For the frequencies <6  GHz, where electromagnetic waves penetrate deeper into tissue, specific energy absorption rate (SAR) in W/kg is introduced denoting the radiation power absorbed by unit mass. Oppositely, in the 6 to 300 GHz range, electromagnetic waves are strongly absorbed by tissue water and, thus, penetrate only the superficial tissues. Therefore, tissue irradiance in ${W/m}^{2}$ (i.e., incident power density per unit area of tissue surface) is used to define the exposure conditions. Considering these two dosimetric quantities, basic limitations of biological object’s exposure to electromagnetic waves^192^ are summarized in Table 3.

**Table 3:** Basic restrictions for the tissue exposure to the electromagnetic waves.

Exposure scenario	Frequency, GHz	Exposure time, min	Whole-body average specific absorption rate, W/kg	Irradiance, ${W/m}^{2}$
Occupational	6–300	≥6	0.4	100
	2–300	30	Not applicable	50
General public	6–300	≥6	0.08	20
	2–300	30	Not applicable	10

In Ref. 196, different energy quantities that measure safety guidelines to protect humans from thermal effects of the MMW and sub-MMW exposure were analyzed. The power density inside tissues correlates with the surface temperature elevations. Since different tissues possess distinct effective dielectric response in the THz range, their complex dielectric permittivity [Eq. (1)] or complex refractive index [Eq. (2)] should be taken into account during estimations of SAR. Methods for the SAR analysis at THz frequencies were developed by several research groups.^42^^,^^197^^,^^198^ In Ref. 199, simulations of a 0.45-THz-wave absorption by the skin revealed that the electromagnetic-wave energy is mostly absorbed in the upper stratum spinosum, whereas the maximal temperature rise is observed in the lower stratum spinosum. The authors showed that the skin temperature can rise beyond the skin injury threshold of 45°C, when the skin is already under the initial heat stress or when a number of radiation sources are applied simultaneously, each within the ICNIRP guidelines.

**Fig. 8:**
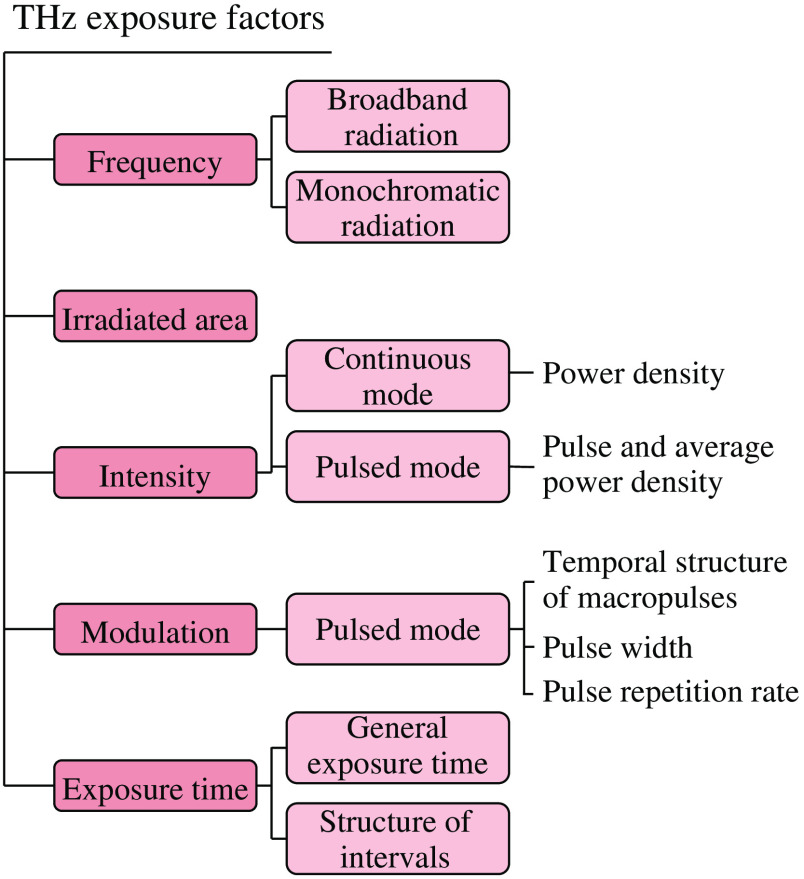
Important technical factors for the experimental studies on THz irradiation. Courtesy of the authors.

For obtaining the reproducible THz-wave effects and adequate dosimetry, the following important conditions should be satisfied during experiments:

1. Technical parameter of the THz emitter (see Fig. 8), conditions and geometry of the THz exposure should be known *a priori*.
2. Cuvette for the THz exposure experiments should be transparent in the THz range.
3. Optical properties n and α of an exposed object should be known *a priori*.
4. THz exposure of an object should be performed in a suitable environment. For example, undisturbed cell growth requires the temperature of ≃37°C, certain humidity, and ≃5% CO content in an ambient atmosphere. Thus, the exposure of cells is performed in an incubator.
5. Adequate controls must be used during the THz exposure:^31^
  • Sham control: reference cells are handled near the exposed ones, but they are not irradiated by THz waves.
  • Temperature control: THz exposure is carried out at the temperatures above the expected thermal heating, for example, 45°C.
  • Positive control: specific agents/substances are applied to activate some function of cells that can be affected by THz waves.
  • Negative control: specific agents/substances are applied to inhibit some function of cells that can be affected by THz waves.
  • Blind control: analysis of the THz exposure effects is performed by a researcher who has no information on what samples were exposed to THz waves and what were used as control group.

All studies of the nonthermal THz-wave effects can be divided into two main categories:

• First one uses low-power THz-wave emitters, which either lead to a small increase in the sample temperature (≤1°C) or do not cause any temperature alterations at all. The results of such THz exposure experiments are compared with control samples that are not irradiated by THz waves but are in the equal ambient conditions with the same temperature background.
• Second one uses powerful THz-wave emitters, which leads to a pronounced heating of a sample and often induces its thermal stress. In such experiments, an exposed sample is compared with the control one, which is heated to the temperature observed for the exposed sample.

When overviewing consistently different THz exposure experiments below, we consider both the temperature conditions of the experiments and the results of heat shock testing only for those experiments, which belong to the second category.

An important point in studying the biological effects of THz waves concerns selection and sustaining the time intervals between the beginning/end of the THz exposure and the analysis. Response of a living organism to any external factor, including the THz radiation, is developed in several stages, including a compensatory stage. The complexity of these stages in each specific case is still impossible to trace since any radiation-induced processes can begin later or finish earlier than some particular features are analyzed. Therefore, such parameter as the time intervals between the beginning/end of the THz exposure and analysis varied to a large extent in different studies. They can range from several minutes to several days or (in some cases) can be not indicated at all. While surveying the research results below, the experiment metrology and these time intervals are not considered comprehensively.

### 4.4 Effects of THz Radiation on the Structural Components of Cells

Considerable amount of data have been accumulated on the THz dielectric spectra of molecular components of cells, such as amino acids,^200^^,^^201^ proteins,^202^^–^^204^ nucleic bases,^205^^,^^206^ and acids,^207^ as well as sugars.^32^^,^^66^ At the same time, very limited knowledge about the nonthermal THz-wave effects on such important molecular components of a living cell exist nowadays.

**Fig. 9:**
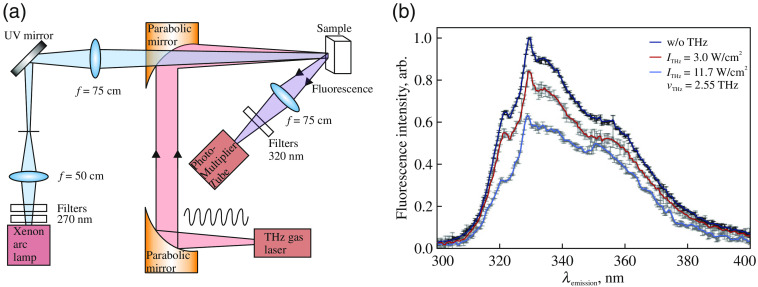
(a) Schematic of the experimental setup. The UV beam at 270 nm was isolated from the broadband light generated by a xenon-UV-enhanced arc lamp to excite a sample. A tunable far-IR gas laser produced CW THz radiation. CW UV and CW THz radiations were combined using a parabolic mirror with a hole that allowed UV radiation to pass through it. The fluorescence signal emitted by a sample was isolated using an interference filter, with the central wavelength of 320 nm and the bandwidth of 10 nm, and, then, collected by a lens and detected by a photomultiplier tube. (b) Fluorescence spectra of Trp pellet sample, either with or without the THz exposure. The frequency of CW THz radiation was 2.55 THz, whereas the THz irradiance was 3.0 and $11.7\;W/{\mathrm{cm}}^{2}$. Adapted from Ref. 208 with the permission of the Taylor and Francis Group.

The effect of CW THz radiation on the fluorescence of the tryptophan (Trp) amino acid and proteins was studied in Ref. 208. A remarkable feature of these experiments is a combination of the THz exposure and the fluorescence spectroscopy in a unitary experimental setup. Such favorable combination allows for observing the THz exposure effects either *in situ* or immediately after the exposure. The experimental setup and related fluorescence spectra are shown in Fig. 9. During the THz exposure, changes in the sample temperature were monitored using a microbolometric camera^209^^,^^210^ and a thermocouple. A 90-s-long exposure of the Trp sample to the CW 2.55 THz radiation, with the irradiance of $11.7\;W/{\mathrm{cm}}^{2}$, caused a decrease in the fluorescence signal by ≃54%.^208^ This drop of the fluorescence signal depends linearly on the THz-radiation intensity, and it closely follows the changes in the sample absorptivity. This points out at the resonant character of the THz-wave–Trp interaction.^208^ For the whey proteins, similar THz exposure leads to the drop of the fluorescence signal by ≃10% and ≃0.26% at the frequencies of 0.2 and 2.55 THz, correspondingly. This exposure simultaneously leads to an increase in the sample temperature by ≃1°C. The control experiments with a simple sample heating confirmed that the observed THz-wave-induced changes are nonthermal in nature, as well as that such nonthermal effects dominate at the lower THz frequencies.

THz exposure of the green fluorescent protein (GFP) demonstrated an opposite dynamics of the fluorescence quenching at 0.2 THz, as well as the fluorescence enhancement by ≃5±0.3% at 2.55 THz while the irradiance was $120\;\mathrm{mW}/{\mathrm{cm}}^{2}$.^208^ Possible mechanism of such a frequency-selective response can be associated with the vibrational resonant coupling between the THz radiation and the protein structure, as explained in the framework of the Fröhlich’s theory.^174^ In Ref. 179, THz exposure of a hen-egg white lysozyme was combined with a highly sensitive x-ray crystallography to visualize low-frequency vibrational modes in the protein structure. It was found that CW 0.4 THz radiation induces a local increase in the electron density in a long α-helix motif consistent with a subtle longitudinal compression of the lysozyme helix. The observed electron density changes can only be explained by a collective excitation of dipole oscillators in the protein, as envisaged by Fröhlich.^174^

In Ref. 211, bovine serum albumin (BSA) was exposed, for 60 min, to the CW 3.67 THz waves with the irradiation of $20\;\mathrm{mW}/{\mathrm{cm}}^{2}$. As the result, changes in the UV and circular dichroism (CD) spectra of the irradiated protein were observed. They were attributed to modifications in the BSA conformation, as was further confirmed by an increase in the Trp fluorescence of BSA^212^ and a twofold decrease in the progesterone binding constant.^211^^,^^212^ In Ref. 213, BSA was exposed, for 60 min, to the pulsed THz radiation with the 0.2- to 1.5-THz spectral bandwidth and the peak irradiance of $10\;\mathrm{mW}/{\mathrm{cm}}^{2}$ (or the average irradiance of $10\;\mathrm{nW}/{\mathrm{cm}}^{2}$), whereas the sample was in the form of thin films on a crystalline ${\mathrm{SiO}}_{2}$ substrate. Then, BSA was dissolved in water and brought into interaction with some adsorbates with high biological relevance, i.e., oxygen, ozone, and nitric oxide. It was found that THz waves caused evident changes in such interactions. Interaction between BSA and oxygen was studied by means of *in situ* spin probing technique. A spin probe was formed directly in solution through the interaction of a diamagnetic dinitrone compound with the reaction sites of BSA, on which oxygen molecules were adsorbed. Quantitative electron paramagnetic resonance spectroscopy demonstrated that the number of reaction sites of the BSA molecule increased by a factor of ≃2 as a result of THz exposure. THz irradiation excites definite collective rotational motions, which partially eliminate steric hindrance for the adsorption of molecular oxygen on the functional groups of BSA.^213^

In other studies, THz exposure of several proteins resulted in the following effects:

• a decrease in the enzymatic activity of alkaline phosphatase;^214^
• an increase in the activity of T7 endonuclease;^215^
• a decrease in the stability of the antigen–antibody complex for dinitrophenol (conjugated with BSA) and mouse monoclonal antibodies against it;^214^
• a dose-dependent change in the activity of alcohol dehydrogenase from yeast, horseradish peroxidase, and BSA.^216^

**Fig. 10:**
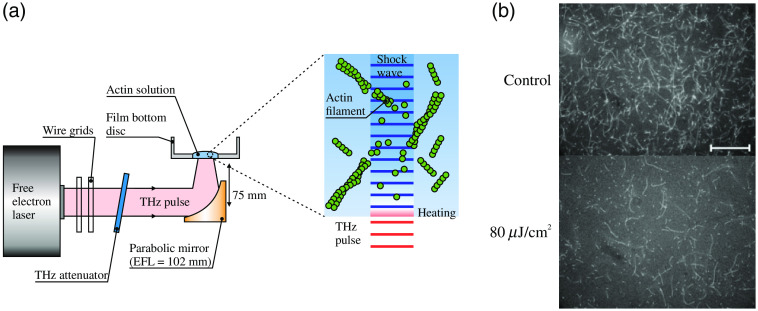
Actin polymerization affected by the THz exposure in an aqueous solution. (a) A schematic of the experimental setup. Actin solution is placed atop of a film bottom dish. THz waves with the frequency of 4.0 THz radiate this dish with a liquid sample from the bottom side. After the THz exposure, a portion of the actin solution is collected for the fluorescence microscopy. (b) Results of the fluorescence microscopy of the actin filaments in control sample (top) and that exposed to THz waves with the irradiance of $80\;\mu J/{\mathrm{cm}}^{2}$ (bottom). Adapted from Ref. 217 with the permission of Springer Nature.

In Ref. 217, modulation of actin polymerization and change in the stability of its filaments, as a result of exposure to intense THz radiation of a free electron laser, were demonstrated; see Fig. 10. At the same time, when actin polymerization was performed under the exposure to the 0.46 THz waves of a gyrotron, the number of actin filaments was increased by a factor of 3.5. In Ref. 218, a change in the conformation (i.e., a decrease in the number of β-sheets and an increase in α-helices) was shown in a five-residue peptide with the amino acid sequence of the calcitonin hormone fragment. In Ref. 216, a reduction in the disordered structure due to an increase of the α-helices and β-sheets was observed as a results of BSA exposure to pulsed THz radiation with the frequency of 3.3 THz and the doses of 1.2 and 3.0 J.

In Ref. 215, THz-wave induced structural changes were also demonstrated for such model systems, as short (tens of bases long) single-stranded DNA with the specified nucleotide sequences. As a result of their exposure to the broadband pulsed THz radiation with the 0.1 to 3.0 THz spectral range, dissembling or interruption of assembling of deterministic secondary thermodynamic DNA structures in an aqueous solution was observed. Such effects were identified by analysis of fluorescent labels and electrophoresis. In turn, for such relatively large molecules as plasmids, there were practically no effects of THz waves on the molecular structure.

In Refs. 186 and 219, THz radiation was found to cause the DNA demethylation. It was shown that molecules of 5-methylcytidine have three major resonance peaks at the frequencies of 1.29, 1.74, and 2.14 THz at a room temperature, whereas molecules of 2’-deoxycytidine did not show any distinct resonances in the 0.4 to 2.5 THz range.^220^ The authors assumed that these THz molecular resonances of 5-methylcytidine are the fingerprints of methylation that are observed at the nucleoside level and can appear at the DNA level.^220^ Then, the frozen aqueous solutions of the normal cellular DNA (293 T cells), the corresponding methylated DNA (methylated 293 T), and the cancer DNA from five types of human cancer cell lines were studied by THz pulsed spectroscopy at the temperature of 253 K. A judiciously design procedure for the THz absorption spectra fitting allowed for isolating the broad absorption peak at ≃1.7  THz, which was similar to the 1.74 THz absorption peak of 5-methylcytidine with slightly shifted central frequency due to an impact of water molecules.^220^^,^^221^ In this way, the authors suggested that THz waves might cause resonant effect on global demethylation and, then, proceeded to the experimental confirmation of this hypothesis.^186^^,^^219^

**Fig. 11:**
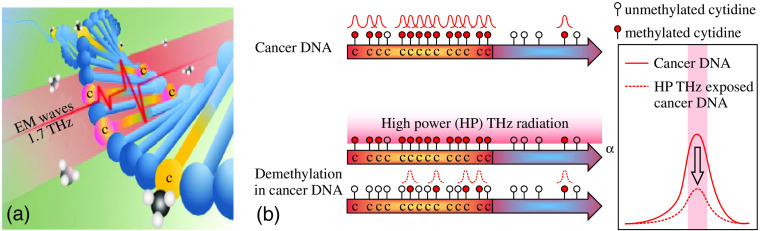
THz-wave-induced DNA demethylation. (a) A scheme of the demethylation by a 1.7-THz resonant exposure of the methylated DNA. (b) Decrease in the amplitude of a resonant THz-wave absorption peak during the demethylation process. Reproduced from Ref. 186 with the permission of Springer Nature.

In Fig. 11, a schematic of the THz-wave-induced demethylation of the methylated DNA is shown. High-power THz emitter was equipped with a bandpass filter, featuring the central frequency of 1.5 THz and the bandwidth of 0.42 THz, aimed at matching the methylated DNA resonance. Magnitude of the resonance peaks observed by the THz pulsed spectrometer decreased after the high-power THz exposure.^186^^,^^219^ For the artificially methylated DNA samples from the human embryonic kidney cells (HEK293T line), demethylation was up to the level of normal values. For the hypermethylated DNA isolated from leukocytes of different cancer lines, it was up to ≃70%. Moreover, for the DNA from HEK293T cells, it was found that the demethylation in genes is observed mainly in the so-called CpG islands, i.e., regions with a high density of the cytosine-guanine dinucleotide sequences.^219^

It is worth noting that, in further studies, Tao et al.^222^ did not observe a 1.6-THz resonance absorption peak of the methylated nucleoside 5-methylcytidine in frozen aqueous solutions. This queries the above-mentioned results of studying the resonant effects of the THz DNA demethylation. In our opinion, this research direction deserves special attention and criticism, whereas further research efforts are necessary for the objective disclosure of THz capabilities to demethylation of DNA.

Parameters of the discussed experiments with exposure of biological molecules to THz waves and their main results are summarized in Tables 4–6. Thus, described experimental studies of the protein and DNA samples show that the main THz-induced effects are associated with changes in the conformation and biological activity of biopolymers. These effects might underlie more complex responses of living systems to THz waves, which are described below.

**Table 4:** Exposure of some biological molecules to THz waves in the CW mode.

Molecules	Frequency, THz	I, $\mathrm{mW}/{\mathrm{cm}}^{2}$	Duration, min	Effects	Refs.
Trp	2.55	3 000	1.5	The fluorescence quenching, 10%	208
		11 700	1.5	The fluorescence quenching, 54%	
Whey proteins	0.2	140	1.5	The fluorescence quenching, 10%	208
	2.55	140	1.5	The fluorescence quenching, 0.26%	
GFP	0.2	120	1.5	The fluorescence quenching, 3%	208
	2.55	120	1.5	The fluorescence enhancement, 5%	
BSA	3.67	20	60	Changes in the UV and CD spectra, the fluorescence enhancement, twofold decrease in the progesterone binding constant	211,212
Alkaline phosphatase	0.1	0.008	90–120	Reduction in enzyme activity	214
Antigen antibody complex	0.1	0.008	90	Decrease of interaction	214
Lysozyme crystals	0.4	62	25 ms	A subtle longitudinal compression of the α-helix	179

**Table 5:** Exposure of some biological molecules to THz waves in the pulsed mode.

Molecules	Frequency (THz)	Pulse duration	Repetition rate	Peak power or irradiance	Duration, min	Effects	Refs.
BSA	0.2–1.5	2–3 ps	∼80 MHz	20 mW	60	Increase the number of reaction sites of BSA	213
BSA	3.3	120 ns	3 Hz	5 mJ	3.3	Increase in the number of β-structure and α-helices; decrease in the binding capacity	216,300
Alcohol dehydrogenase	3.3	120 ns	3 Hz	5 mJ	2.2	Nonlinear change in enzymatic activity	216
T7 endonuclease	0.1–3.0	∼1 ps	2.5 kHz	0.4 mJ	10	Increase in catalytic activity	215
G-actin	0.46	10 ms	1 Hz	$5.7\;\mathrm{mJ}/{\mathrm{cm}}^{2}$	20	The number of actin filaments was increased by 3.5-fold	301
Plasmid DNA	0.1–3.0	∼1 ps	2.5 kHz	0.4 mJ	10	No effect	215
dsDNA of up to ∼20 bp	0.1–3.0	∼1 ps	2.5 kHz	0.4 mJ	10	Completely dissociated dsDNA	215
The genomic DNA samples	1.5±0.21	—	—	$2.4\;\mathrm{mW}/{\mathrm{cm}}^{2}$	30	Global demethylation	186
HEK293T DNA	1.7±0.28	—	—	$2.4\;\mathrm{mW}/{\mathrm{cm}}^{2}$	30	THz demethylation mainly affected cytosine in CpGI (53%), where CpG sites were abundant	219

**Table 6:** Exposure of some biological molecules to THz waves in the complex mode.

Molecules	Frequency (THz)	Micropulse duration	Macro-pulse duration	Repetition rate of macropulses	Peak power	Duration (min)	Effects	Refs.
Fibril of calcitonin peptide	4.05, 3.74	10 ps	4 μs	5 Hz	∼1 mJ (macro)	30	Decrease of β-sheet; increase α-helix, turn, and others	218
G-actin	4±1	5 ps	100 pulses per macro-	5 Hz	80, 160, 250 μJ (micro)	30	Decrease of actin filaments	217

### 4.5 Effects of THz Radiation on Cells and Cell Cultures

Next, we overview the THz-wave effects on the separate cells and different cell cultures. For this aim, different types of cells are analyzed separately, with an emphasis on the blood, skin, cornea, nerve, and stem cells.

#### 4.5.1 Blood cells

Considerable amount of data on the THz optical properties of blood and its components was accumulated up-to-date.^2^^,^^223^^–^^225^ THz-pulsed spectroscopy measurements, with the typical range of 0.1 to 3.2 THz, the average beam power of $10^{-7}$ to $10^{-9}\;W$, and the measurement duration of 1 to 5 min, did not lead to any changes in the spectral and morphological properties of blood cells.^226^ Such low-power THz-pulsed radiation satisfies the discussed ICNIRP guidelines and is reportedly harmless for biological systems.^41^

At the same time, a number of research groups demonstrated considerable effects of THz waves on blood cells. In Ref. 227, exposure, for 3 h, of human red blood cells (RBC) to the CW radiation of BWO, with the frequencies of 0.18 to 0.33 THz and the irradiance of $3\;\mathrm{mW}/{\mathrm{cm}}^{2}$, decreases their osmotic resistance, which was attributed to the release of hemoglobin from erythrocytes. In Ref. 228, when human RBCs were exposed, for 1 h, to the CW THz radiation with the frequency of 3.68 THz and the irradiance of $40\;\mathrm{mW}/{\mathrm{cm}}^{2}$, the hemoglobin release from erythrocytes with an addition of water in the ratio of 1:2 increased by seven times, as compared with the nonirradiated erythrocytes. From Ref. 46, it follows that viability of rat RBCs exposed, for 1 h, to pulse THz radiation, with the spectral range of 0.1 to 1.75 THz, the pulse repetition rate of 76 MHz, the pulse duration of 1 ps, and the peak pulse power of 8.5 mW, decreases to a greater extent than in control samples when erythrocytes are placed in the 0.54% to 0.48% NaCl solutions. The cell viability was assessed using trypan blue dye, which penetrates into cells when its membrane is broken.

In Ref. 229, human RBC were exposed, for 5, 10, 15, 20, and 25 s, to the Novosibirsk free electron laser radiation, with the spectral range of 2.05 to 2.31 THz, the pulse repetition rate of 5.6 MHz, the pulse duration of 120 ps, the peak pulse power of <1  MW, and the average irradiance of 8 to $10\;W/{\mathrm{cm}}^{2}$. A 5-s-long THz exposure did not lead to pronounced changes in the morphology of cells and did not reduce their aggregation. In turn, increase of the exposure time to 10 to 15 s changed the cell morphology (spherocytosis) and decreased the number of erythrocytes in the aggregates (as a linear function of the exposure duration). A 25-s-long exposure led to the destruction of cell membranes and the lysis of erythrocytes. The observed effects were attributed to the passage of powerful ultrasonic waves that were induced by the THz pulses at the laser pulse repetition rate of 5.6 MHz, through the irradiated medium. As a control, heating of erythrocytes to body temperature and their exposure to ultrasound were used, where such effect was not observed. These results indicated a THz-wave-induced change in the cell membrane permeability.

**Fig. 12:**
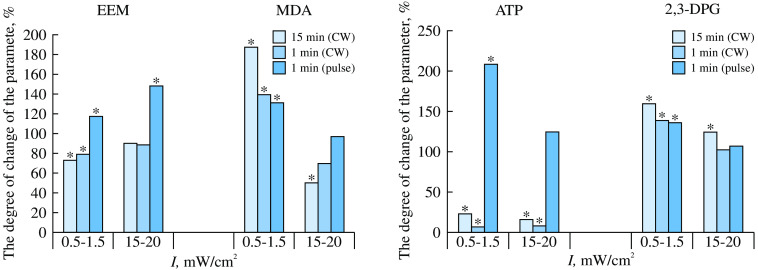
Changes in blood cell parameters after the THz exposure with different irradiances, where 100% corresponds to the control level. EEM, erythrocytes electrophoretic mobility; MDA, malondialdehyde; ATP, adenosine triphosphate; 2,3-DPG, 2,3-diphosphoglycerate. The asterisk * indicates statistically significant differences (p<0.05) as compared with the control group. Adapted from Ref. 230 with the permission of Springer Nature.

An important aspect of studying living organisms under the THz exposure is an evaluation of the antioxidant status of cells, particularly, of erythrocytes. In Ref. 230, RBCs were exposed to the CW THz radiation, with the frequency of 0.263 THz and the irradiance of 0.5 to $20\;\mathrm{mW}/{\mathrm{cm}}^{2}$, for 1 and 15 min, and to the pulsed THz radiation, with the pulse repetition rate of 2.5 Hz, the pulse duration of 200 ms, and the average irradiance of 0.1 to $20\;\mathrm{mW}/{\mathrm{cm}}^{2}$, for 1 min. As a result of the THz exposure, the intensity of lipid peroxidation (malondialdehyde concentration) in erythrocytes increased as compared with the nonirradiated control sample, whereas the observed effect depends on the exposure duration. In both regimes, a nonlinearity of the effect was observed, as shown in Fig. 12. At the irradiances of 0.5 to $1.5\;\mathrm{mW}/{\mathrm{cm}}^{2}$, oxidation was more pronounced than at 5 to $20\;\mathrm{mW}/{\mathrm{cm}}^{2}$. Such effects may indicate both induction of the oxidative stress upon THz irradiation and activation of the response of the cell antioxidant systems.^230^ Other parameters were also altered after such an exposure. The electrophoretic mobility of cells decreased after the CW THz exposure and, conversely, increased after the pulsed one, regardless of the irradiance value. In turn, the metabolic parameters, such as ATP and 2, 3 diphosphoglycerate concentrations, depended on both the exposure regime and irradiance.^230^

In Ref. 231, safety limits of blood leukocytes exposure to THz waves were studied, using several pulsed THz emitters and the method of DNA comets.^232^ The picosecond pulsed THz radiation, with the spectral range of 0.1 to 6.5 THz, the average irradiance of up to $200\;\mu W/{\mathrm{cm}}^{2}$, and the exposure duration of 20 min, did not induce any DNA damage in blood leukocytes while heating of thus exposed sample did not exceed 1°C. In Refs. 233–235, no effects of the human white blood cell exposure, for 20 min, to the pulsed THz radiation, with the central frequencies of 0.12 and 0.13 THz, the pulse repetition rates of 21.1 and 73.7 kHz, the pulse duration of 50 ps, and the average irradiance of 30 to $230\;\mu W/{\mathrm{cm}}^{2}$, were observed, including the absence of the direct DNA damage, the chromosome apparatus changes, and the alteration of cell cycle kinetics. However, in Ref. 235, under certain conditions of cells’ exposure in a THz resonant cavity, some signs of direct DNA damage appeared.

In Ref. 228, exposure, for 30 and 90 min, of the human total blood lymphocytes to the CW THz radiation, with the frequency of 3.68 THz and the irradiance of $40\;\mathrm{mW}/{\mathrm{cm}}^{2}$, led to a decrease in the number of viable cells, which was more pronounced at high exposure time. It is worth noting that such THz exposure causes an increased proliferative activity of the surviving cells. In Ref. 236, similar cells were exposed, for 1, 2, and 24 h, to the CW THz radiation, with the frequency of 0.1 THz and the irradiance of $31\;\mu W/{\mathrm{cm}}^{2}$, which was followed by studying four chromosomes (1, 10, 11, and 17) in cells during division. The observed results revealed an increase in aneuploidy of two chromosomes at 2- and 24-h exposure and changes in centromere replication of three and all four chromosomes at 2- and 24-h exposure, respectively. Thus, such a long-term (≥1  h) THz exposure induces significant genomic instability.

In Ref. 237, human T-lymphocytes (Jurkat cell line) were exposed, for 5, 10, 20, 30, and 40 min, to the CW THz radiation with the frequency of 2.52 THz and the irradiance of $227\;\mu W/{\mathrm{cm}}^{2}$. As a result, almost no changes in the lymphocytes survival were observed in the case of a short-term exposures (<20  min). In turn, an exponential increase of the lymphocytes’ death was observed for the long-term THz exposure (≥20  min) due to both apoptosis and necrosis of cells. Finally, for the 40-min-long THz exposure, up to 80% of cells died.

Studies of the global gene expression profile remain the most representative in the THz exposure technologies. For example, in Refs. 238–240, human T-lymphocytes (Jurkat cell line) were exposed to the 2.52-THz CW radiation with the irradiance of $636\;\mathrm{mW}/{\mathrm{cm}}^{2}$ for 30, 40, and 50 min, accompanied by an increase in the sample temperature from 37°C to 44°C. Thus, irradiated cells were then compared with the control group heated to the same temperatures. Pilot measurements revealed that a 40-min-long exposure to THz waves causes a change in the expression of 628 genes, whereas the 40-min-long bulk heating changes expression of 556 genes.^238^ Further measurements appeared to be even more illustrative, demonstrating a change in expression of 597 and 725 genes, respectively, with an overlap of only 61 genes. Completely different signaling and metabolic pathways were identified for the THz exposure and heating. There were 30 and 16 pathways for the THz exposure and heating, respectively.^172^^,^^240^ Quite interesting nonlinear effects were observed, i.e., some genes of the potassium and calcium channel proteins showed increased activity when exposed for 40 min, as compared with the 30- and 50-min-long exposures.^238^ An additional analysis of individual genes indicated that, although stress genes are activated during the THz exposure, this is not typical for all studied genes and is less pronounced as compared with a simple heating.^239^

Overall, studies of blood cell exposure to THz waves revealed the following biotropic effects:

• an increase in the cell membrane permeability;
• influence on various aspects of metabolism (including the oxidative stress), morphology, proliferation, and aggregation activity;
• gene and cytotoxic effects.

In some reactions, fingerprints of the compensatory mechanisms were found. Further extensive studies of the global gene expression profiling that captures many aspects of cellular life deserve special attention. THz effects are often highly constructive and sometimes nonlinear in relation to changes of the THz exposure parameters. The discussed effects of blood cell exposure to THz waves in different regimes are summarized in Table 7. Notice that no effects are observed at low exposure intensity (bottom six lines of the table); whereas various effects occur with the increased exposure (top 12 lines of the table).

**Table 7:** Exposure of blood cells to THz waves in the different modes.

Cells	Frequency (THz)	Type	Irradiance ($\mathrm{mW}/{\mathrm{cm}}^{2}$)	Duration (min)	Effects	Refs.
Human RBCs	2.05 to 2.31	Pulsed	10000	0.42	Lysis	229
Jurkat cells	2.52	CW	636	30 to 50	Activation of genes of intracellular signal transduction pathways	238
Jurkat cells	2.52	CW	636	240	Increase expression of genes of heat shock proteins, cell growth factors, antiinflam matory cytokines	239
Jurkat cells	2.52	CW	227	20 to 40	Cell death due to both apoptosis and necrosis of cells	240
Human RBCs	3.68	CW	40	60	Decreased osmotic resistance	228
Human blood lymphocytes	3.68	CW	40	30, 90	Decreased cell viability	228
Rat RBCs	0.263	CW	0.5 to 20	1, 15	Electrophoretic mobility of cells decreases, destructive changes of the cell morphofunctional state	230
Rat RBCs	0.263	Pulsed	0.1 to 20	1	Activation of the metabolic processes	230
Human blood lymphocytes	0.13	Pulsed	2, 5	20	DNA damage	235
Human RBCs	0.18, 0.33	CW	3	180	Decreased osmotic resistance	227
Human blood lymphocytes	0.1	CW	0.031	120, 1440	Increased aneuploidy of chromosomes 11 and 17 during cell division	236
Rat RBCs	0.1–1.75	Pulsed	0.01	60	Increased rate of hypoosmotic hemolysis	46
ICNIRP standard	0.002 to 0.3	—	1	30	None	40
Human blood lymphocytes	0.1	CW	0.013	20	None	236
Human blood leukocytes	0.5 to 6.5	Pulsed	0.008	20	None	231
Human blood leukocytes	0.1 to 2.0	Pulsed	0.125	20	None	231
Human blood leukocytes	0.1 to 1.0	Pulsed	0.2	20	None	231
Human blood lymphocytes	0.12 to 0.13	Pulsed	0.03 to 0.25	20	None	233

#### 4.5.2 Skin and cornea cells

In 2015, the Scientific Committee on Emerging and Newly Identified Health Risks published an opinion on the potential health effects of electromagnetic exposure. Given the expected increase in the use of THz technologies, it was recommended to pay special attention to studying the THz-wave effects on the skin, with an emphasis on the long-term low-intense exposure, and on the cornea, with an emphasis on the short-term high-intense exposure.^241^ Therefore, fibroblasts and keratinocytes of the skin, as well as epithelial cells of the cornea, are the most extensively studied cell types in the THz exposure technologies.

In Refs. 242 and 243, human dermal fibroblasts (HDF cell line) were exposed, for 5, 10, 20, 40, and 80 min, to the CW THz radiation with the frequency of 2.52 THz and the irradiance of $85\;\mathrm{mW}/{\mathrm{cm}}^{2}$. This exposure was accompanied by heating of a samples from 37°C to 40°C, therefore, the control sample was heated to 40°C and studied as a reference. Additional genotoxic control samples were exposed, for 3 min, to the CW UV radiation, with the wavelength of 254 nm and the average power of 38 W. A 5- to 20-min-long THz exposure did not lead to a decrease in the number of living cells, whereas longer exposure slightly reduced it. At the same time, some increase in the cell proliferation was observed. As a results of the THz exposure, an increase in expression was noted for genes of the heat shock proteins, however, it was almost equal to that of conventional heating. No enhancement in the expression of DNA sensing and repair genes, which was observed under the UV irradiation, was found after the THz exposure. Overall, no considerable changes at a cellular and molecular level were identified.^242^^,^^243^ At a more intense exposure of the same cells to the THz-wave source with the irradiance of $227\;\mathrm{mW}/{\mathrm{cm}}^{2}$ and the duration varying from few seconds to 2 min, the death of fibroblasts was observed after only the 12-s-long exposure. Finally, as a result of 1- and 2-min-long THz exposure, activation of the genes of some inflammatory cytokines was revealed.^242^

In Refs. 244 and 245, when human skin fibroblasts (HFFF2 and HDF cell lines) were exposed, for 20 min, to the broadband pulse THz radiation, with the frequency of 0.1 to 0.15 THz, the pulse repetition rate of 21.1 to 26.3 kHz, the pulse duration of 50 ps, and the average irradiance of $0.4\;\mathrm{mW}/{\mathrm{cm}}^{2}$, authors observed aneuploidy effects, such as an increase in actin polymerization, chromosomal malsegregation, and micronucleus induction. Meanwhile, no signs of the DNA damage, such as expression of the corresponding proteins, phosphorylation of H2AX histone, and repair telomere length modulation, were noticed. Also, no THz-wave effects on the cell growth and survival, including changes in the prosurvival signaling proteins, were revealed. Thus, there was a clear aneugenic rather than clastogenic effect of THz radiation.^244^^,^^245^

In Ref. 246, HDFs were exposed, for 20 min, to the CW THz radiation, with the frequency of 0.14 THz and the irradiance of 35 to $354\;\mathrm{mW}/{\mathrm{cm}}^{2}$. Such parameters as proliferative activity, wound closure percentage, and the level of nitric oxide production in the irradiated cells were estimated. No tangible changes in the examined parameters were revealed. Therefore, low-frequency THz radiation does not affect the functional activity of the HDFs.^246^

In Ref. 247, human skin fibroblasts (NB1RGB cell line) and human corneal epithelial cells (HCE-T cell line) were exposed, for 3, 70, and 94 h, to the CW THz radiation, with the tunable output frequency in the range of 0.07 to 0.3 THz and the irradiance of 0.4 to $1.3\;\mu W/{\mathrm{cm}}^{2}$. THz exposure at different frequencies did not reveal any changes in proliferation, morphology, and cell activity, as well as no signs of cytotoxicity.^247^ Exposure of the same cells, for 3, 70, and 94 h, at separate frequencies in the range of 0.3 to 0.6 THz with the irradiance of $<1\;\mu W/{\mathrm{cm}}^{2}$ also did not cause any effects on proliferation, survival, and cell morphology of both cell types.^248^

In Ref. 249, human corneal epithelial cells (HCE-T cell line) and human retinal pigment epithelial cells (ARPE-19 cell line) were exposed to the broadband pulsed THz radiation of synchrotron with a ≃0.5  THz cut-off frequency, a 1 kHz pulse repetition rate, a 2-ps pulse duration, and a $0.85-2.25\;\mathrm{kW}/{\mathrm{cm}}^{2}$ peak irradiance (or a $0.14-0.37\;\mathrm{mW}/{\mathrm{cm}}^{2}$ average irradiance), and different exposure terms in the range of 140 to 230 min were examined. Results showed no signs of cytotoxicity, as well as no changes in the cell morphology and proliferation. It was suggested that cells, cultured and maintained under ideal standard conditions, are capable of compensating the THz-wave effects even in the case of such high exposure intensities.^249^ Negative data were also obtained on the HCE-T cells, exposed to the 0.12-THz CW waves with the irradiance of $5\;\mathrm{mW}/{\mathrm{cm}}^{2}$, and the exposure duration of 24 h. There was no statistically significant increase in genotoxicity, morphological changes, and alterations in the heat shock protein expression.^171^

In Ref. 250, human keratinocytes (NHK cell line) and human corneal epithelial cells (HCE-T cell line) were exposed to the pulsed THz radiation, with the central frequency of 0.14 THz, the pulse repetition rate of 50 kHz, the pulse duration of 80 ns, the peak irradiance of 24 to $62\;\mathrm{mW}/{\mathrm{cm}}^{2}$ (the average irradiance of 0.1 to $0.25\;\mathrm{mW}/{\mathrm{cm}}^{2}$), for the different exposure durations ranging from 10 min to 24 h. The effects of such THz exposure were then compared with those of the UVA (the 315 to 400 nm wavelenths) and UVB (280 to 315 nm) exposures, as well as the 43°C heat shock treatment. No considerable changes in cell activity (viability and proliferation) and differentiation were detected in cases of a single THz exposure (with the duration of up to 24 h) and a double one (with the 48-h-long time interval between the 24-h-long exposure cycles). THz waves did not induce a stress response, such as changes in the glutathione and the heat shock protein 70 levels. At the same time, all studied parameters of cells were sensitive to the UVB exposure and the heat shock.^250^

In Ref. 251, human skin fibroblasts (HDF cell line) and keratinocytes (HaCaT cell line) were exposed, for 2, 8, and 24 h, to the CW THz radiation, with the frequency of 0.106 THz and the irradiance of 0.04 to $2\;\mathrm{mW}/{\mathrm{cm}}^{2}$. Although the authors considered THz irradiance of cells either below or above the aforementioned safety limits, they did not find any changes in proliferation rate and abnormalities in the genetic apparatus, such as increased micronucleus formation, formation of strand breaks, or alkali-labile sites in DNA.^251^ Similar negative results were obtained in Ref. 252, using the same cells, but another THz emitters with the output frequencies of 0.38 and 2.52 THz and the irradiance in the range of 0.03 to $0.9\;\mathrm{mW}/{\mathrm{cm}}^{2}$, whereas the exposure durations were 2 and 8 h.^252^

**Fig. 13:**
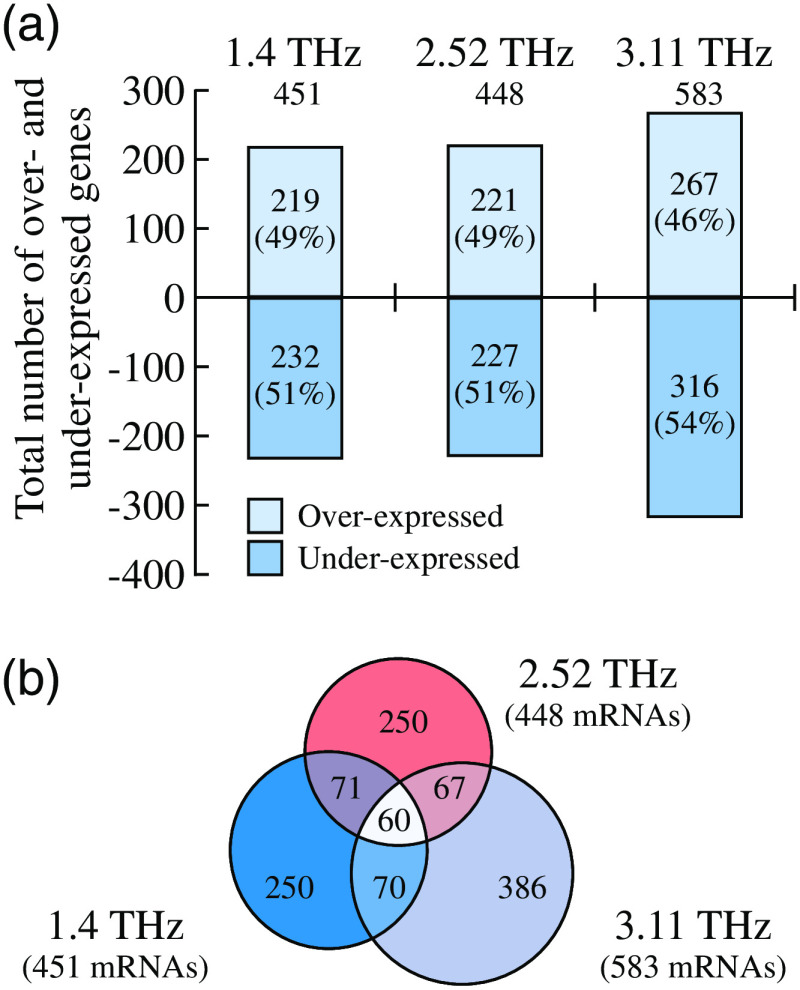
Gene expression profiles of the human epidermal keratinocytes exposed to the THz radiation at different frequencies. (a) Total number of over- and underexpressed messenger RNAs (mRNA) at the 1.4, 2.52, and 3.11 THz exposures. (b) A Venn diagram indicating the number of unique and common differently expressed mRNAs at each THz exposure. Reprinted from Ref. 253 with the permission of SPIE.

The authors of Ref. 253 irradiated human epidermal keratinocytes (HEK001 cell line), for 20 min, by the CW THz radiation with the three distinct frequencies of 1.4, 2.52, and 3.11 THz and the irradiance of $44\;\mathrm{mW}/{\mathrm{cm}}^{2}$. Viability of cells and expression of hyperthermic genes were not altered at such exposure conditions. Analysis of the total gene expression revealed 451, 448, and 583 differently expressed genes under exposure to the 1.4, 2.52, and 3.11 THz waves, respectively. More than 50% of these genes in each group were unique, whereas <25% in one group appeared to be common for all three regimes of the THz exposure. Bioinformatic analysis revealed activation of 6, 17, and 12 canonical metabolic and signaling pathways for the 1.4, 2.52, and 3.11 THz exposure, respectively. These pathways were also unique for the considered exposure conditions, except for the extracellular signal regulated kinase 5, as shown in Fig. 13.

In Ref. 254, human skin keratinocytes from donors were exposed, for 10, 20, and 30 min, to the radiation of the two broadband pulse THz emitters. The first one operated in the frequency range of 0.2 to 3.0 THz, possessed the pulse repetition rate of 80 MHz and the pulse duration of 20 to 30 ps, and provided the average irradiance of $0.009\;\mathrm{mW}/{\mathrm{cm}}^{2}$. The second one features the following set of characteristics: 0.1 to 2.7 THz, 250 kHz, 250 fs, and $9\;\mathrm{mW}/{\mathrm{cm}}^{2}$. As a result of these experiments at both low and high intensities, THz radiation did not affect the cell activity (in general) and did not inhibit their capacity to differentiate, even despite the expected variability in the activity of the primary cells isolated from different donors.^254^

**Fig. 14:**
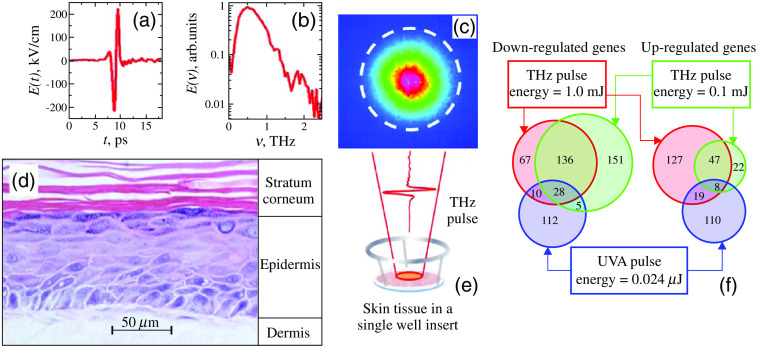
Effects of the intense THz pulses on artificial human skin tissues model EpiDermFT. (a), (b) Time-dependent electric field of the THz pulse E(t), as well as its Fourier spectrum E(ν), respectively. (c) Intensity of the THz focal spot ${\left|E\right|}^{2}$. (d) Histology of an EpiDermFT tissue model. (e) Schematic of the THz exposure, where the EpiDermFT tissue model is mounted at the focal plane. (f) Venn diagrams summarizing the differently expressed genes in the EpiDermFT tissue model, as a result of exposure to either 1.0 mJ THz wave or 0.14 mJ UVA radiation. Adapted from an open-access Ref. 33 with the permission of Springer Nature.

As shown in Fig. 14, HDFs and epidermal keratinocytes of the full thickness artificial human skin tissues model were exposed, for 10 min, to the broadband pulsed THz radiation with the frequencies of 0.1 to 2.0 THz, the pulse repetition rate of 1 kHz, the pulse duration of 1.7 ps, and the average irradiances of 5.7 and $57\;\mathrm{mW}/{\mathrm{cm}}^{2}$.^33^^,^^255^ As a control, these cells were also exposed, for 2 min, to the pulse UVA radiation with the pulse repetition rate of 1 kHz, the pulse duration of 50 fs, the central wavelengths of 400 nm, and the pulse energy of 0.080  μJ. Total gene expression analysis revealed a change in the expression of 397 and 442 genes, as a result of the low- and high-intense THz exposures, respectively, against 293 genes, as a result of the UV exposure. THz radiation selectively reduced the expression of genes associated with apoptosis and skin diseases, such as psoriasis and atopic dermatitis. With regard to the potential risk of carcinogenesis, a dual effect was observed since both anticancer and cancer-promoting genes were activated. Activity of the same genes, under the UV exposure, has a different dynamics than THz. THz exposure showed signs of DNA damage, along with an activation of the DNA damage repair mechanisms, such as histone H2AX phosphorylation and increase in the levels of some proteins. In general, the cellular response to THz radiation is significantly different from the UV-induced one.^33^^,^^255^

When similar object was exposed, for 10 min, to broadband pulsed radiation with quite close parameters (the frequency range of 0.1 to 3.0 THz, the pulse repetition rate of 1 kHz, the pulse duration of 1 ps, the peak irradiance of $74\;\mathrm{MW}/{\mathrm{cm}}^{2}$, or the average irradiance of $74\;\mathrm{mW}/{\mathrm{cm}}^{2}$) in Refs. 256–258, 1681 genes changed their expression, among which 1088 and 593 genes were down- and upregulated, respectively. Bioinformatic analysis showed that through the observed changes in the activity of many of these genes, the processes of initiation, maintenance, and progression of a cancer are suppressed; suppression of the glioma pathway is especially well indicated. Despite the fact that these predictions are based on observations in the skin tissue models, the gene-level mechanisms responsible for the negative perturbation are genes that encode for proteins involved in the calcium and mitogen-activated protein kinase signaling; these are ubiquitous and well-conserved across many different cell types, including skin and neural cells.^258^ Moreover, the authors observed explicit activation of an inflammatory response and suppression of a promitotic signaling, including the suppression of cellular functions, such as cell division, differentiation, motility, and apoptosis. Existence of an energy threshold for the observed effects is quite important: namely, at lower THz radiation intensities (the pulse energy of <1.5  μJ or the electric field strength of <187  kV/cm), no significant changes were observed, as compared with the intense THz exposure (2.4  μJ and 240  kV/cm), that caused the genetic changes.^256^^–^^258^ Among the observed genomic response, special attention was paid to the THz-wave effect on calcium signaling pathway due to its general significance in biological regulation. An additional study of the differential gene expression profiles, in the case of the five different THz exposure irradiances in the range of 0.6 to $47\;\mathrm{MW}/{\mathrm{cm}}^{2}$, revealed suppression of this pathway by THz radiation and enhancement of this effect with the exposure intensity.^259^

**Fig. 15:**
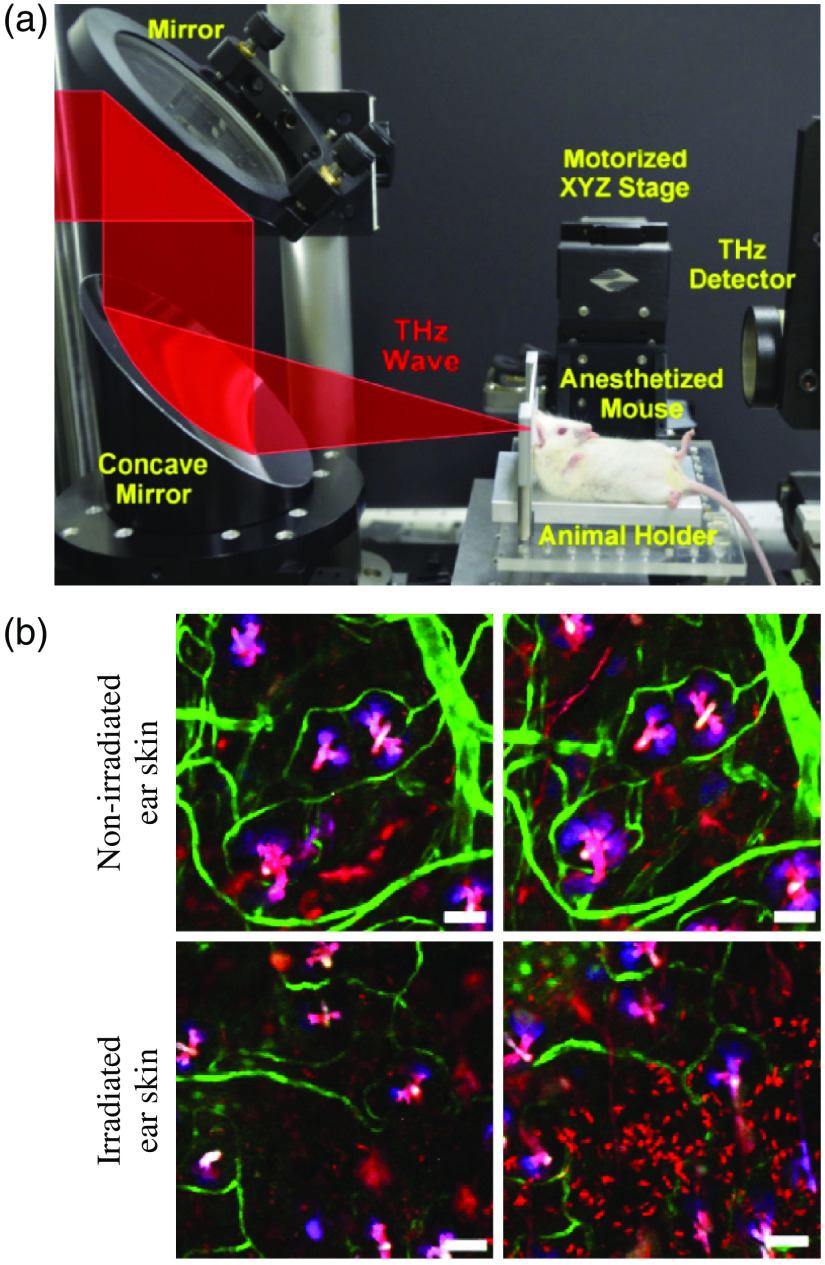
THz exposure of the mouse ear skin *in vivo*. (a) A photo of the THz setup. A living anesthetized mouse is placed on an animal holder that is fixed at a motorized 3D translation stage. (b) Distribution of neutrophils in the mouse ear skin before and after the THz exposure: Gr-1 + neutrophil (in red), Tie2 + blood vessel (green), and autofluorescent hair follicle (magenta). Adapted from Ref. 261 with the permission of the Optical Society of America.

In Ref. 260, mouse keratinocytes of dorsal skin were exposed *in vivo*, for 1 h, to the broadband pulsed THz radiation, with a 0.1- to 2.5-THz spectral range, a 1-kHz pulse repetition rate, a 310-fs pulse duration, and a $0.032\text{-}\mathrm{mW}/{\mathrm{cm}}^{2}$ average irradiance. Analysis of the genome-wide expression profile showed a change in the activity of 149 genes involved in the processes of tissue growth and healing, organogenesis, and cell migration. Further bioinformatic analysis indicated a difference in the pattern of gene expression, as a result of the THz exposure, as compared to the UV and neutron ones. Relying on additional studies of individual genes, the authors concluded that general expression gene pattern, induced by THz radiation, is analogous to that by wound stimulus.^260^ In Ref. 261, mouse ear skin *in vivo* was exposed, for 30 min, to the pulsed radiation with a 2.7-THz central frequency, a 3-Hz pulse repetition rate, a 4-μs pulse duration, and a $260\text{-}\mathrm{mW}/{\mathrm{cm}}^{2}$ average irradiance. Schematic of this exposure is shown in Fig. 15. The observed results indicated an acute inflammatory response in the skin (the infiltration of neutrophils), whereas IR camera detected no notable THz-wave-induced change in the skin temperature.^261^ The results showed that such an acute inflammatory response can be initiated without structural disruption of the skin by THz radiation. THz exposure of living tissue in the *in vivo* conditions can possibly trigger various unexpected dynamic responses, which can not be mimicked appropriately in the simplified *in vitro* conditions.^261^

Thereby, the overviewed research demonstrated the main reaction of the skin and cornea cells on THz exposure. These cells act in multicellular organisms as the primary barrier that perceives THz waves as a factor of the external environment and protects against it. THz-wave-driven changes at cellular and molecular levels were revealed, both clearly negative, such as the DNA damage and aneuploidy effects, and relatively harmless, such as the increased cell proliferation. Many results of the THz exposure, including the ones observed in terms of geno- and cytotoxic effects, turned out to be negative, emphasizing that the considered regimes of THz exposure were lower than biologically significant thresholds. A comparison of the observed THz effects with other factors, such as the UV exposure and temperature stress, and with the whole-genome gene expression profiling aided by the bioinformatics, confirmed variability and specificity of the cellular response to THz waves.

Tables 8 and 9 show some remarkable results, both positive and negative, of the skin and cornea cells exposure to THz radiation. One should notice that CW exposure overall causes no damage when using the considered small-to-moderate THz exposure intensities. In turn, due to much higher peak power, pulsed THz exposure can cause DNA damage and changes in gene expression. Thus, considerable attention should be paid to determine safe limits of such pulsed exposure to skin and cornea, with an observed rapid developments of THz technology and, particularly, THz wireless communications.

**Table 8:** Effects of CW THz radiation on the skin and cornea cells.

Cells	Frequency (THz)	Irradiance (${\mathrm{mW}/\mathrm{cm}}^{2}$)	Duration (min)	Effects	Refs.
Human skin fibroblasts	2.52	84.8	40, 80	Number of viable cells decreased by 10%; enhanced proliferation; no DNA damage	243
Human skin fibroblasts	2.52	227	1, 2	Activation of proinflammatory cytokines and stress response	237
Human epidermal keratinocytes (HEK001 cell line)	1.4, 2.52, 3.11	44	20	The activation of metabolic and signaling pathways specific for each frequency	253
ICNIRP Standard	0.002 to 0.3	1	30	None	40
Human skin fibroblasts	0.14	35 to 354	20	No effect on proliferative activity, wound closure percentage, and the level of nitric oxide production	246
Human skin fibroblasts (NB1RGB cell line)	0.07 to 0.3	<0.001	180, 4200, 5760	Not any effects on proliferation, survival and cell morphology	247
Human corneal epithelial cells (HCE-T cell line)	0.07 to 0.3	<0.001	180, 4200, 5760	Not any effects on proliferation, survival and cell morphology	247
Human corneal epithelial cells (HCE-T cell line)	0.12	5	1440	No statistically significant increase in genotoxicity, morphological changes, and alterations in the heat shock protein expression	171
HaCaT and HDF cells	0.106	0.04 to 2.0	120, 480, 1440	No induction of DNA strand breaks or chromosomal damage	251
HaCaT and HDF cells	0.38, 2.52	0.03 to 0.6, 0.05 to 0.9	120, 480	No induction of DNA strand breaks or chromosomal damage	252

**Table 9:** Effects of pulse THz radiation on the skin and cornea cells.

Cells	Frequency (THz)	Irradiance (${\mathrm{mW}/\mathrm{cm}}^{2}$)	Duration (min)	Effects	Refs.
Artificial human skin	0.1 to 2.0	5.7, 57	10	Formation of DNA double strand breaks, activates DNA damage response	33,255
Artificial human skin	0.1 to 3.0	0.9 to 74	10	Suppression of the *Ras* signaling and calcium signaling pathways	259
Mouse ear skin *in vivo*	0.1 to 2.5	0.032	60	Activation of genes included in tissue growth	260
Mouse ear skin *in vivo*	2.7	260	30	An acute inflammatory response was initiated without structural disruption of the skin	261
Human fibroblast culture	0.1 to 0.15	0.4	20	No DNA damage; enhanced actin polymerization	244
Human corneal epithelial cells (HCE-T cell line)	0.5	0.14 to 0.37	140 to 230	No signs of cytotoxicity, no changes in the cell morphology and proliferation	249
Human keratinocytes (NHK cell line)	0.14	0.1 to 0.25	10 to 1440	No considerable changes in cell viability, proliferation and differentiation	250
Primary human keratinocytes	0.2 to 3.0, 0.1 to 2.7	0.009, 9	10, 20, 30	No effect on cell activity and differentiation	254

#### 4.5.3 Nerve cells

**Fig. 16:**
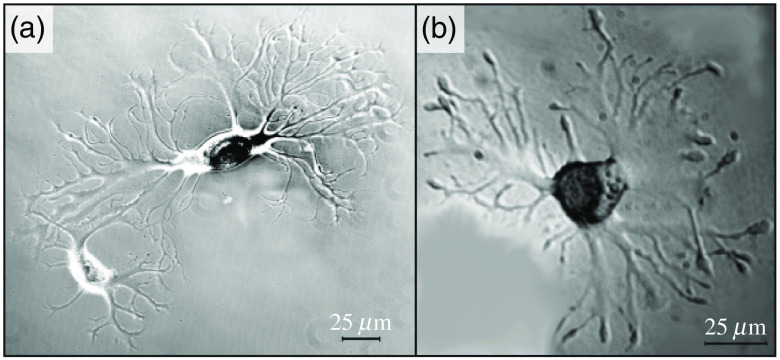
Effect of the 3.68-THz CW radiation on the isolated neurons at the stage of the neural network formation. (a) Microscopy of the unaltered neural network. (b) Neural network formed after the THz exposure. In panel (b), disturbances in the neurites’ growth zone, and cessation of their further growth, including the disordering in the interneuronic links, are observed. Courtesy of the authors.

In Ref. 262, effects of the CW laser THz waves on the isolated neurons of the supraesophageal galglion of the *Lymnea stagnalis* were investigated at 0.71, 1.63, 2.45, 2.56, 3.68, and 4.28 THz, whereas the irradiance and the exposure duration were 2 to $20\;\mathrm{mW}/{\mathrm{cm}}^{2}$ and 1 h. At 0.71 THz, the adhesive characteristics of membranes were altered, and the contacts between the nerve cells and a substrate were disturbed. At 3.68 THz, the structural changes occurred in the somatic membrane, axons, and growth cone. The effects were delayed and manifested themselves only 40 to 50 h after the exposure. During this period, redistribution of pigment granules occurred, and the membrane cytocortical layer became heterogeneous. Then, anomalous outgrowth-like structures with the arbitrary orientation grew, whereas classical neurites did not form.^262^ In the same experiment, the response of cell membrane to the THz exposure was not identical at the different stages of the neural network formation. The aforementioned effects occurred at the initial stage of the neural network regeneration, i.e., before formation of the neuron outgrowths. Other phenomena were observed in neurons (with the outgrowths already formed) at the stage of the neuron network formation, namely, the disturbance in the neurites’ growth zone and the cessation of their further growth, which caused disorders in the interneuron links’ formation,^262^^,^^263^ as shown in Fig. 16.

In Ref. 263, the same biological object, namely, the isolated neurons of the *Lymnea stagnalis*, was exposed, for 1 min, to the pulsed THz radiation with the central frequencies of 2.1 and 2.3 THz, the pulse repetition rate of 5.6 to 11.2 MHz, the pulse duration of 30 to 100 ps, and the average irradiance of 0.3 to $30\;\mathrm{mW}/{\mathrm{cm}}^{2}$. Exposure at 2.3 THz and $30\;\mathrm{mW}/{\mathrm{cm}}^{2}$ caused a gradual decrease in the membrane potential, accompanied by the morphological disorders of membrane and intracellular structures, as well as by the cell death within 2 h after the exposure. At $3\;\mathrm{mW}/{\mathrm{cm}}^{2}$, cell death occurred within 3 h after the exposure; whereas at $0.3\;\mathrm{mW}/{\mathrm{cm}}^{2}$, part of cells remained vital, and the number of vital cells stabilized within 2 h after the exposure. However, when resorting to 2.1 THz, even with the minimal irradiance of $0.3\;\mathrm{mW}/{\mathrm{cm}}^{2}$, no viable cells were detected in 1 h after the exposure.^263^

**Fig. 17:**
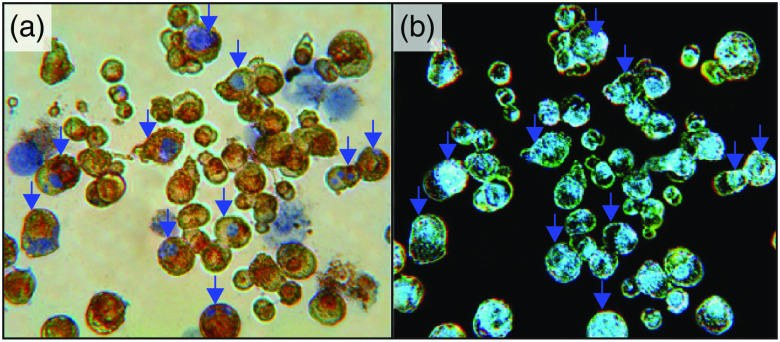
Neurons after the 2.3-THz CW exposure with the irradiance of $0.5\;\mathrm{mW}/{\mathrm{cm}}^{2}$ and the duration of 1 min. (a) White light microscopy of the trypan blue-stained cells. (b) Fluorescence microscopy of the trypan blue + BCECF-AM-stained cells. Reprinted from Ref. 263 with the permission of NSU publishing.

In Refs. 263 and 264, barrier properties of the neuron membrane were studied after the exposure to THz radiation of a free electron laser possessing the frequency of 2.3 THz and irradiance in the range of 0.5 to $20\;\mathrm{mW}/{\mathrm{cm}}^{2}$ (see Fig. 17). Such exposure causes a dose-dependent nonspecific permeability of the cell membrane that was uncovered by studying the vital dye trypan blue transmission through the membrane into the cytoplasm. The dye was distributed nonuniformly in cytoplasm, being localized in separate regions. This effect is reportedly due to the THz-wave-induced formation of hydrophilic pores in the cell membrane. It is reversible, because the membrane potential and functional reactions of cells return to the normal values within 1 day after the exposure.^263^^,^^264^ Exposure of cells to the 2.0-THz THz waves with similar parameters did not cause significant changes in the majority of cells. Only sole neurons were colored uniformly, and their membrane potential was decreased or equal to zero, whereas their amount did not differ from the reference values.^264^ The control of membrane recovery after the disturbance was carried out using the BCECF-AM dye (7′-bis(2-carboxyethyl)-5(6)-carboxyfluorescein acetoxymethyl ester). This dye can penetrate through the undamaged plasmatic membrane, and it is transformed by the intracellular esterases of living neurons into its fluorescent form BCECF. Fluorescence of noncolored cells and some cells that captured tripan blue was discovered. Membrane of such cells can be recovered after disturbance, and they can retain fluorescence probes inside.^263^

To test the assumption concerning the THz-wave-induced formation of hydrophilic lipid pores and to reveal the underlying mechanisms before the THz exposure, antioxidants were introduced into the salt solution surrounding neurons, along with a dye (lucifer yellow) that does not penetrate through the intact membranes. It was found that phenol antioxidant histochrom significantly reduces the penetration of the dye into the cell. This may indicate that hydrophilic pores are formed in the cell membrane due to the free radical processes that can be blocked by antioxidants.^265^ Thus, THz radiation may cause reversible disturbance of the membrane barrier properties, serving as an inductor of the biologically active compounds’ delivery into cells. In turn, antioxidants may be applied to manage this process, providing protection from unfavorable THz-wave effects.^265^

In Ref. 266, a dose-dependent cytotoxic effect was demonstrated during exposure for 1 to 5 min of the rat glial cells (C6 cell line) to the CW THz radiation of BWO, with the output frequencies in the range of 0.12 to 0.18 THz and the irradiance of $3.2\;\mathrm{mW}/{\mathrm{cm}}^{2}$. A relative number of apoptotic cells increased while sample heating did not exceed 0.1°C. This study posed a problem of possible biological harm caused by such common CW THz-wave sources as BWOs.^266^

Reference 267 demonstrated an impact of the broadband pulsed THz radiation, with the frequencies in the range of 0.05 to 2.0 THz, the irradiances of 0.5, 5, and $50\;\mu W/{\mathrm{cm}}^{2}$, and the exposure duration of 3 min, on the neurite growth in the sensory ganglia of 10- to 12-day chicken embryos. An increase in the stimulating effect by 24% was observed at the lowest power density of $0.5\;\mu W/{\mathrm{cm}}^{2}$. At the same time, at higher intensities of 5 and $50\;\mu W/{\mathrm{cm}}^{2}$, no notable changes were found. These observations highlighted nonlinear THz-wave effects in relation to the electromagnetic-beam power.

**Fig. 18:**
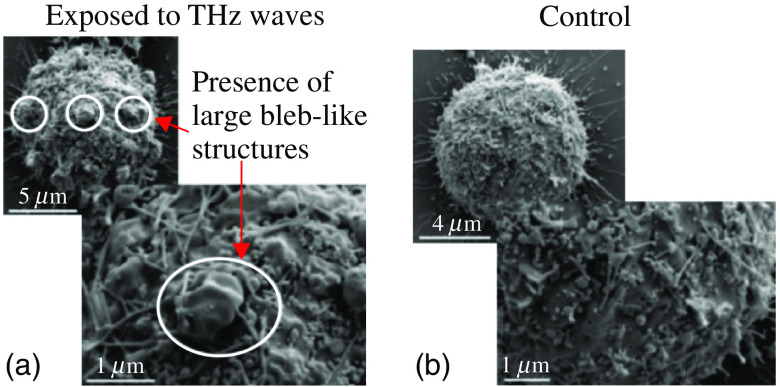
Scanning electron microscopy of the PheoChromocytoma (PC12) cells after 10-min-long THz exposure. As a result of the THz exposure, formation of blebs on the PC12 cells was demonstrated, as highlighted by white circles in (a); whereas blebs were not found in the untreated PC12 cells, as evident from (b). Adapted from Ref. 268 with the permission of MDPI.

In Ref. 268, the neuron-like pheochromocytoma (PC12) cells were exposed, for 10 min, to the 0.3- to 19.5-THz radiation of a synchrotron. During such exposure, the average temperature of the sample was 25.24±0.37°C. High-resolution scanning electron microscopy confirmed permeabilization of the cell membrane. For this aim, translocation of silica nanoparticles into the PC12 cells was visualized. Analysis of the microscopy data revealed formation of atypically large (up to 1  μm) blebs on the surface of PC12 cells exposed to THz waves, as shown in Fig. 18. Significant differences between the metabolic activity of the THz-wave-treated PC12 cells and of the control ones were not found. However, a higher population of the THz-treated PC12 cells responded to the nerve growth factor by extending longer neurites as compared with the untreated PC12 cells.^268^

Exposure to the 0.06-THz CW radiation of the pyramidal rat neurons was studied in Ref. 269. For 1-min-long THz exposure at low irradiances (40 to $840\;\mathrm{nW}/{\mathrm{cm}}^{2}$), considerable changes in the neuronal firing rate and plasma membrane properties were notable. After resorting to higher irradiances (100 to $600\;\mu W/{\mathrm{cm}}^{2}$), dose-dependent increase in the plasma membrane permeability of the intact segmental ganglia of the adult leech (particularly, in the studied Retzius neurons) was observed.^270^ In Ref. 271, THz exposure, for 1 min, of individual neurons in the leech midbody ganglia at even higher irradiances (1 to $4\;\mathrm{mW}/{\mathrm{cm}}^{2}$) revealed different effects of THz waves and equivalent thermal heating on narrowing of the action potential half-width and the firing rate. Possible explanation of this effect is the interaction between the THz wave and the neuronal plasma membrane. In Ref. 272, Romanenko et al. extended their studies by examining the direct effect of 0.06 THz radiation with higher power density (80 to $170\;\mathrm{mW}/{\mathrm{cm}}^{2}$) on sensory neurons from the leech, namely, the thermosensitive nociceptor. Both experiments and simulations showed a notable decrease in the voltage threshold of the action potential formation, as compared with the thermal heating. This effect was attributed to the sensitization of the transient potential of the vanilloid 1-like receptor in the leech nociceptor.

In Ref. 273, the cultured primary hippocampal neurons were exposed, for 20 min, to the CW source (Terasense Group Inc.), with the frequency of 0.1 THz and the irradiance of $33\;\mathrm{mW}/{\mathrm{cm}}^{2}$. The cells were cultured in the 37°C constant temperature incubator and, then, irradiated from the bottom side of the culture dish. RNA-sequencing was performed to identify the expression of 29 genes, as a result of the THz exposure. No considerable difference in the temperature between the culture medium of the sham group and the radiated group was observed. The expression levels of several heat shock proteins genes were not significantly upregulated by the THz exposure. The neuron cells did not undergo serious apoptosis response as well. The authors concluded that THz waves can affect various biomolecule interactions, such as binding of GTPase, phospholipid, tropomyosin, BMP receptor, and long-chain fatty acid, as well as regulate the synapse function and calcium signaling pathway. The upregulation of free intracellular calcium, observed *ex vivo*, along with the correlated morphological changes in the leech ganglia neuron were also demonstrated at 0.06 THz with the power of 100 mW.^274^

The discussed effects of the neuron cells’ exposure to THz waves in CW mode are summarized in Tables 10 and 11. Cytotoxicity effects were observed during a number of experiments on exposure of cells and cell models to THz waves with different parameters. THz-wave-induced changes in the structure and functions of the cytoplasmic membrane, including the distortions of the membrane potential, are evident. The processes of nerve tissue regeneration act as an additional factor that modulates the THz-wave effects. In general, THz exposure of nerve cells paves the ways to the development of methods aimed at the noninvasive and selective action on the molecular and cellular regulatory mechanisms.

**Table 10:** Effects of CW THz radiation on the neurons cells.

Cells	Frequency (THz)	Irradiance (${\mathrm{mW}/\mathrm{cm}}^{2}$)	Duration (min)	Effects	Refs.
Glial cells	0.12 to 0.18	3.2	1	Number of apoptotic cells increased 1.5-fold	266
Glial cells	0.12 to 0.18	3.2	5	Number of apoptotic cells increased 2.4-fold	266
Neurons of *L. stagnalis*	0.71	10–20	60	Alteration of adhesive properties of neuron membrane	262,263
Neurons of *L. stagnalis*	1.63, 2.45, 2.56	2–20	60	None	262,263
Neurons of *L. stagnalis*	3.68	2–5	60	None	262,263
Neurons of *L. stagnalis*	3.68	10–20	60	Alteration of neuron membrane and formation of the interneuron connection (structural changes of the somatic membrane, axons and growth cone)	262,263
Neurons of *L. stagnalis*	4.28	2 to 20	60	None	262,263
The pyramidal rat neurons	0.06	$4\cdot 10^{-5}$ to $84\cdot 10^{-5}$	1	Considerable changes in the neuronal firing rate and membrane	269
Retzius neurons of leech	0.06	0.1 to 0.6	1	Dose-dependent increase in the plasma membrane permeability	270
Neurons of leech	0.06	1 to 4	1	Narrowing of the action potential half-width	271
Thermosensitive nociceptor of leech	0.06	80 to 170	1	Notable decrease in the voltage threshold of the action potential formation	272
Primary hippocampal neurons	0.1	33	20	Changing various biomolecule interactions as well as regulate the synapse function and calcium signaling pathway	273
The leech ganglia neuron	0.06	100	1	Upregulation of free intracellular calcium and morphological changes	274

**Table 11:** Effects of pulsed THz radiation on the neurons cells.

Cells	Frequency (THz)	Irradiance (${\mathrm{mW}/\mathrm{cm}}^{2}$)	Duration (min)	Effects	Refs.
Neurons of *L. stagnalis*	2.1	0.3	1	Cell death after 60 min	263
Neurons of *L. stagnalis*	2.3	0.3	1	Membrane changes	263,264
Neurons of *L. stagnalis*	2.3	3	1	Cell death after 120 min	263,264
Neurons of *L. stagnalis*	2.3	30	1	Cell death after 180 min	263,264
Neurons of *L. stagnalis*	2.3	2 to 20	0.6	Reversible membrane permeability	265
The sensory ganglia	0.05 to 2.0	0.0005	3	Stimulating effect by 24%	267
The sensory ganglia	0.05 to 2.0	0.005, 0.05	3	None	267
PC12 cells	0.3 to 19.5	—	10	Formation of atypically large blebs on the surface of cells	268

#### 4.5.4 Stem cells

Stem cells are extremely sensitive to the environmental stimuli. Therefore, they can be considered as a favorable model for studying the effects of biological system exposure to weak electromagnetic fields including the THz waves. Pluripotency is a unique attribute of stem cells. Thus, a maintenance of the pluripotent state or, vice versa, an increase in the rate of spontaneous or induced differentiation are important features to be investigated, from the point of view of both their high sensitivity and their practical significance.

In Ref. 275, human embryonic stem cells (hESM01 cell line) were exposed, for 1 h, to the pulsed THz radiation, with the central frequency of 2.3 THz, the peak irradiance of $4\;\mathrm{kW}/{\mathrm{cm}}^{2}$ (the average irradiance of $≃0.14\;W/{\mathrm{cm}}^{2}$). The whole genome analysis revealed the altered expression of 73 genes, whereas only 1 of those genes (namely, PRDM14, which suppresses differentiation) was specific to the pluripotent state. In total, 74% of the THz-sensitive protein-coding genes belong to the class of ubiquitously expressed genes. Bioinformatic analysis of the affected genes revealed 15 functional classes that were mostly related to mitochondria. Additional studies did not demonstrate signs of genotoxicity (structural chromosomal aberrations and phosphorylation of histone H2AX), morphological signs of spontaneous differentiation, and any effect on the mitotic index. In Ref. 249, similar human embryonic stem cells (hES07 cell line) were exposed to the broadband pulsed THz radiation, with the cut-off frequency of ≃0.5  THz, the pulse repetition rate of 1 kHz, the pulse duration of 2 ps, the peak irradiances in the range of $1.75\;\mathrm{kW}/{\mathrm{cm}}^{2}$ (the average irradiance of 0.21 to $0.29\;\mathrm{mW}/{\mathrm{cm}}^{2}$). The exposure duration varied in the range of 120 to 375 min. The authors observed no changes in the cell morphology, attachment, differentiation, and proliferation.

In Ref. 188, bone marrow mesenchymal stem cells were exposed, for 25 min, to the 0.1- to 4.5-THz pulsed THz radiation with a 75-μJ pulse energy and a ≃10-MV/cm field strength. Cell viability analysis was carried out 24 h after the exposure, for which the fluorescent staining with propidium iodide and Hoechst dyes was applied. This study did not reveal any increase in the number of dead cells, as compared with the control group.

Human-induced pluripotent stem cells (hiPSCs) were exposed to the pulsed THz radiation with the central frequency of 0.8 THz and the electric field of 0.5  MV/cm in Ref. 276. The estimated temperature rise was only 0.3 mK, which could not trigger to the expression of heat shock proteins. The genes, strongly affected by THz irradiation, were regulated by zinc-finger proteins. The authors supposed that gene expression was induced nonthermally by the electric field of the THz pulse, which might be caused by the movement of zinc ions within the cell compartments.

Mouse mesenchymal stem cells (MSC cell line) were studied in Ref. 277. Cells were preliminarily induced to differentiate toward adipocytes. They were exposed, for 2, 4, 6, and 9 h, to the broadband pulsed THz waves with the frequency range of 1 to 30 THz (the maximal intensity is centered at ≃10 THz), the pulse repetition rate of 1 kHz, the pulse duration of 35 fs, the peak power of ≃30  MW, and the average irradiance of $≃1\;\mathrm{mW}/{\mathrm{cm}}^{2}$. Analysis of the genome-wide expression profile showed a clearly visible change in the profile only after a 9-h exposure; namely, 2204 genes changed their activity. Additional analysis of individual genes and visually increased accumulation of the lipid-like droplets in the cellular cytoplasm justified acceleration of the cells’ differentiation into adipocytes under prolonged irradiation. Further studies that involved a 12-h-long THz exposure of cells to the radiation of the same source as well as their 2-h-long THz exposure to the CW radiation (the frequency of 2.52 THz and the power of <150  mW) revealed changes in the activity of 381, 122, and 236 genes at 12-h broadband pulsed, 2-h broadband pulsed, and 2-h CW exposure regimes, respectively. Finally, it was found that a 2-h-long THz exposure (regardless of the applied THz sources) affects genes transcriptionally active in pluripotent stem cells.^278^^,^^279^ The authors note that the transcriptional response to THz irradiation indirectly indicates manifestation of gene-specific intrinsic double-stranded DNA breathing dynamics, and the THz-field effects are most likely at the level of DNA transcription.^277^^–^^279^

There is still a quite small amount of investigation in the area of stem cell exposure to THz waves. The observed effects, both at the cellular and genetic levels, can be divided into two categories: general and stem-cell-specific. Effects from the first category, such as cyto- and genotoxicity, have not yet been found against the background of changes in the expression of many genes. Among the effects from the second category, available data allow us to consider THz radiation as a potential tool for noncontact control of gene expression and modulation of cell differentiation.

### 4.6 Effects of THz Waves on Fluorescent Cellular Biosensors

**Fig. 19:**
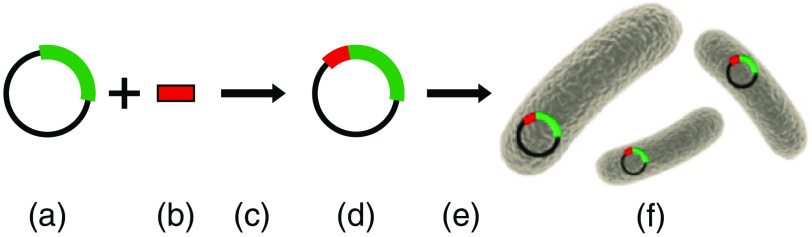
The general scheme of the biosensor development: (a) a base vector with a fluorescence protein gene, (b) a sensory gene promoter, (c) an assembly, (d) a hybrid genetic construct, (e) an introduction of the resulting construct into cells, and (f) biosensors. Courtesy of D.S. Serdyukov.

Fluorescent cellular biosensors, described in this section, generally have a form of cells with artificial genetic constructs, which include a promoter of a sensor gene (it reacts to the effect of one or another chemical or physical factor) and a reporter gene of fluorescence protein (its activity is regulated by the promoter of the sensor gene).^280^ The development of such biosensors includes assembly of the hybrid genetic constructs and their introduction into bacterial cells, as shown in Fig. 19.

When exposed to the investigated factor, the promoter of a sensor gene activates the reporter gene and, accordingly, the production of the fluorescence protein, thus, leading to a well-detectable fluorescent signal. The biosensory approach to study THz bioeffects makes it possible to trace the THz response of a gene, the promoter of which was used in the assembly of the genetic construct.

**Fig. 20:**
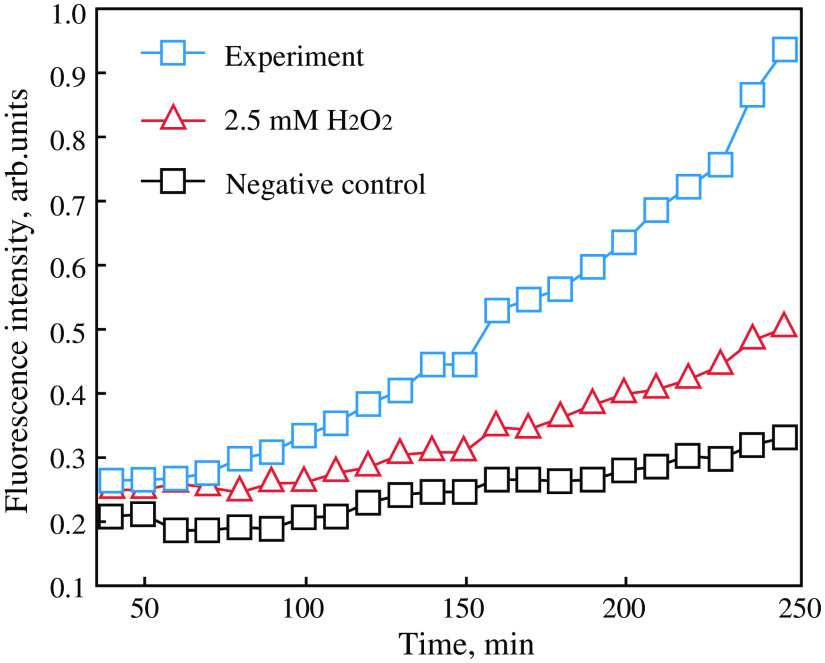
Fluorescence dynamics of the *E. coli* biosensor cells with the promoter of a sensor gene *katG* after their 15-min-long exposure to 2.0 THz radiation, as compared with the bulk heating to 37°C (negative control) and with the exposure to hydrogen peroxide. Adapted from Ref. 281 with the permission of John Wiley and Sons, Inc.

*E. coli* biosensor cells with the promoter of a sensor gene of catalase (*katG* gene) were exposed, for 5, 10, and 15 min, to pulse THz irradiation, with the central frequencies of 1.50, 2.00, and 2.31 THz, the pulse repetition rate of 2.8 to 11.2 MHz, the pulse duration of 50 ps, the peak power of 1 MW, or the average irradiance of $1.4\;W/{\mathrm{cm}}^{2}$. THz radiation was intense enough to heat the samples up to 33°C to 37°C; therefore, the control samples were subjected to the bulk heating at 37°C. In addition, a positive control was applied; namely, exposure to the hydrogen peroxide (a typical inductor for the *katG* gene) and two additional controls: heat (42°C for 5 min) and cold (30°C for 15 min) shock. As a result, it was shown that THz radiation activates this biosensor (i.e., the catalase gene in the *E. coli* genome) at all studied THz frequencies, and the effect appears to be dose-dependent. There was no activation at 5-min-long exposure as well as after the heat/cold shock. At the same time, activation was observed in some experiments at 10-min-long exposure and in all experiments at 15-min-long exposure. Figure 20 shows that the effect of THz waves turned out to be more pronounced than that of a positive control; it persisted for more than 4 h after the exposure, which is equal to about eight life cycles of *E. coli*.

At the same time, radiation did not affect cell survival.^281^ Later, the activity of *E. coli* biosensors with the promoter of a sensor gene *copA* (involved in the homeostasis of copper ions) and the promoter of a sensor gene *emrR* (involved in multidrug resistance) was studied using the same THz source with the central frequency of 2.31 THz and exposure duration of 15 min. The similar temperature conditions were applied. There were also corresponding positive controls that involved exposure to copper (II) sulfate and salicylic acid. As a result, the *emrR* promoter was not activated during the THz exposure, but there was a prolonged activation (by analogy with *katG*) of the *copA* promoter, but in less extent in comparison with the positive control; heat shock had no effect.^282^ Long-term THz-dependent responses of *E. coli* stress systems associated with oxidative stress and copper ion metabolism were demonstrated. The absence of a reaction of the antibiotic resistance system emphasizes the specificity of the cellular response.^281^^–^^283^

Recently, similar *E. coli* biosensor technology was applied to study the activities of promoters that regulate the production of transcription factors MatA, YdeO, ChbR, and TdcR.^284^^,^^285^ Being exposed, for 15 and 30 min, to the intense-pulsed THz radiation, with the frequency of 2.31 THz, the pulse repetition rate of 5.6 MHz, the pulse duration of 100 ps, and the average irradiance of $≃140\;\mathrm{mW}/{\mathrm{cm}}^{2}$, the samples were heated up to the temperatures of 35°C to 37°C, and all four biosensors were activated, as compared with the bulk heating at 37°C. However, when exposed, for 15 and 30 min, to the low-intensity 0.14-THz CW radiation with the irradiance of $2\;\mathrm{mW}/{\mathrm{cm}}^{2}$, the samples were heated up to ≃26°C (which is ≤1°C above an ambient room temperature), and only three biosensors were activated, displaying the biosynthesis of YdeO, ChbR, and TdcR. In almost all of these cases, a dose-dependent effect was observed, i.e., the activation was more pronounced (or was only observed) at 30-min-long exposure, as compared to the 15-min-long one. In addition, as an example of THz-dependent activation of the *tdcR* gene, the significant influence of the type of vessel for irradiation and the composition of the nutrient medium was shown. Chemical exposure (five different toxins separately) or heat shock (heating up to 42°C) did not cause the activation. Thus, four THz-sensitive biosensors were obtained, which serve as indicators of various cellular functions: biofilm development (MatA), response to various stress (YdeO), uptake and metabolism of chitobiose (ChbR), and transport and metabolism of threonine and serine (TdcR).

Thereby, the described research results justify that nowadays biosensors are considered as a promising platform for research and monitoring of the THz anthropogenic factors.

## 5 Discussions

Evidently, during the last few decades, large amounts of data have been accumulated regarding biological effects of THz waves. Sometimes, we observed contradicting results of the THz exposure, which poses important problems of adequate designing, planning, and performing of the THz exposure experiments. As described earlier, only a combination of accurate knowledge about the exposure parameters, a number of control measurements (tests), and maintenance of the suitable ambient environment can lead to reproducible and verified experimental data.

Despite a considerable interest paid to the biological effects of THz waves, further research is required for the development of safe limits of THz waves. Obviously, such safe limits should account for not only the thermal THz-wave effects (as the common ICNIRP standards do)^40^^,^^192^^,^^193^ but also for the nonthermal. Development of the THz dosimetry is of crucial importance for biomedical applications of THz technology in such demanding branches as label-free diagnosis of malignant and benign neoplasms,^3^^,^^4^^,^^8^^–^^16^ sensing of glycated tissues and blood in context of diabetes diagnosis,^1^^,^^19^^–^^21^ determining the degree of traumatic injuries^22^ and viability^30^ of tissues, and even emerging methods of single cells, microorganisms, bacteria, and viruses sensing.^286^^–^^291^ Also, THz dosimetry is of crucial importance for the rapidly developing 6G wireless communications that will reportedly span sub-THz and THz frequencies.^292^ There is no doubt that such a wide range of THz technology applications, which involve interaction between THz waves and different biological systems, would stimulate the development of THz safe limits in the nearest future.

Studies of the THz-wave biological effects involving modern methods of cytology, genetics, and molecular biology can uncover THz therapeutic avenue. As mentioned above, THz waves are capable of regulating the gene expression, changing the membrane permeability, and DNA demethylation. Being adequately studied and regulated, such a versatile impact of THz waves on living cell opens a variety of THz technology applications in medical therapy of cancers, inflammatory, and neurodegenerative deceases. Finally, we notice that development of such THz therapeutic applications would require further progress in THz components, including highly efficient uncooled CW and pulsed THz emitters,^6^^,^^7^^,^^126^^–^^128^^,^^130^^,^^132^^–^^135^ elements of bulk (open space) and fiber/waveguide optics^293^^–^^299^ aimed at the THz-wave delivery to the hardly accessible tissues and internal organs. Such elements are still to be developed.

## 6 Conclusion

Recent research results in the area of THz-wave effects on biological systems of the different organization levels, such as biomolecules, cells, and organism, were discussed. Despite the considerable data accumulated in this demanding research direction, we still possess quite limited knowledge about biological effects of THz waves. Further research and engineering efforts are required to develop adequate safe limits of THz-wave exposure and to objectively uncover strengths and weaknesses of THz technology in different branches of medial therapy. Thus, this review summarizes up-to-date knowledge in the area of cell exposure to THz radiation and paves the ways to the THz dosimetry and therapeutic avenues.

## Code, Data, and Materials Availability

All data generated or analyzed during this study are included in this article.
